# Targeting dendritic cells to accelerate T-cell activation overcomes a bottleneck in tuberculosis vaccine efficacy

**DOI:** 10.1038/ncomms13894

**Published:** 2016-12-22

**Authors:** Kristin L. Griffiths, Mushtaq Ahmed, Shibali Das, Radha Gopal, William Horne, Terry D. Connell, Kelly D. Moynihan, Jay K. Kolls, Darrell J. Irvine, Maxim N. Artyomov, Javier Rangel-Moreno, Shabaana A. Khader

**Affiliations:** 1 Department of Molecular Microbiology, Washington University in St Louis, 660 S Euclid Avenue, St Louis, Missouri 63110, USA; 2 Richard King Mellon Foundation Institute for Pediatric Research, Children's Hospital of Pittsburgh of UPMC, 4401 Penn Avenue, Pittsburgh, Pennsylvania 15224, USA; 3 Witebsky Center for Microbial Pathogenesis & Immunology, Department of Microbiology & Immunology, University at Buffalo, 3435 Main Street, Buffalo, New York 14214, USA; 4 Department of Biological Engineering, Massachusetts Institute of Technology, 500 Main Street, Cambridge, Massachusetts 02139, USA; 5 Department of Materials Science and Engineering, Massachusetts Institute of Technology, 77 Massachusetts Avenue, Cambridge, Massachusetts 02139, USA; 6 Division of Immunobiology, Washington University in St Louis, 660 S Euclid Avenue, St Louis, Missouri 63110, USA; 7 Department of Medicine, Division of Allergy, Immunology and Rheumatology, University of Rochester Medical Center, 601 Elmwood Avenue, Rochester, New York 14642, USA

## Abstract

The development of a tuberculosis (TB) vaccine that induces sterilizing immunity to *Mycobacterium tuberculosis* infection has been elusive. Absence of sterilizing immunity induced by TB vaccines may be due to delayed activation of mucosal dendritic cells (DCs), and subsequent delay in antigen presentation and activation of vaccine-induced CD4^+^ T-cell responses. Here we show that pulmonary delivery of activated *M. tuberculosis* antigen-primed DCs into vaccinated mice, at the time of *M. tuberculosis* exposure, can overcome the delay in accumulation of vaccine-induced CD4^+^ T-cell responses. In addition, activating endogenous host CD103^+^ DCs and the CD40–CD40L pathway can similarly induce rapid accumulation of vaccine-induced lung CD4^+^ T-cell responses and limit early *M. tuberculosis* growth. Thus, our study provides proof of concept that targeting mucosal DCs can accelerate vaccine-induced T-cell responses on *M. tuberculosis* infection, and provide insights to overcome bottlenecks in TB vaccine efficacy.

## Abstract (web summary)

A delay in T cell responses is postulated as a possible explanation for the limited efficacy of vaccines against tuberculosis. Here the authors demonstrate this T-cell block and remove it by activating endogenous dendritic cells or delivering activated dendritic cells to the lungs, enhancing immunity of mice to *Mycobacterium tuberculosis*.

Tuberculosis (TB) is a leading cause of death by infection^1^. TB is caused by aerosol exposure to the intracellular bacterium *Mycobacterium tuberculosis* (*Mtb*), leading to either latent disease or active pulmonary disease. The only currently licensed vaccine against TB is *Mycobacterium bovis* Bacille Calmette–Guerin (BCG). Although BCG vaccination is effective against childhood forms of TB^1^, and in decreasing childhood TB morbidity^1^, it provides variable efficacy against adult pulmonary TB. Thus, over the past two decades, concerted efforts have been made to develop new vaccines for TB that will provide improved protection on *Mtb* exposure. Modern candidate TB vaccines have focussed on induction of T-cell responses, primarily CD4^+^ T cells producing interferon gamma (IFNγ)^2^. More recently, an important role for mucosal interleukin 17A (IL-17A) in vaccine-induced protection against TB disease has been shown^3^,^4^,^5^,^6^. Thus, induction of lung-resident IL-17A-producing CD4^+^ T-cell populations by TB vaccines is also being explored^2^,^4^,^5^,^7^. Despite these efforts, most TB vaccines reduce the burden of lung *Mtb* by ∼0.5–1.0 log in animal challenge models^3^,^8^,^9^,^10^,^11^. Recombinant live mycobacterial vaccines confer improved protection (∼2 log reduction), when compared with subunit and virally vectored TB vaccines. Examples of recombinant vaccines include the recombinant *Mycobacterium smegmatis* vaccine, which induces sterilizing immunity in the liver, but not the lung^12^; recombinant BCG over-expressing *Listeria monocytogenes* listeriolysin and lacking urease C^11^,^13^; and the recombinant *Mtb* vaccine lacking *PhoP*^14^. Other work has highlighted the benefit of administering recombinant *Mtb* vaccines mucosally, showing that macaques vaccinated with the attenuated *Mtb* mutant lacking *SigH* had sterile protection in some TB lesions^15^. Although these results are promising, considerable challenges are associated with the design and implementation of a recombinant *Mtb* vaccine to be delivered mucosally via the lungs, particularly in light of the TB–HIV co-epidemic. Thus, it is critical to fully understand the early events occurring in the vaccinated *Mtb*-infected lung.

After *Mtb* infection of naive mice, accumulation of activated lung CD4^+^ T cells is delayed, occurring between 14 and 21 days post *Mtb* infection (dpi)^16^,^17^. This delay is thought to be due to *Mtb*-mediated inhibition of early antigen presentation by antigen-presenting cells (APCs)^18^,^19^,^20^. Even in the presence of an existing *Mtb*-specific vaccine-induced CD4^+^ T-cell population in the lung, CD4^+^ T-memory cells do not accumulate in the lung until 12–14 dpi^3^,^6^,^21^. Priming of T-cell responses following primary *Mtb* infection requires trafficking of *Mtb*-infected DCs from the lungs to the lymph nodes (LNs)^20^,^22^. Furthermore, *Mtb*-infected DCs do not efficiently present antigen directly to *Mtb*-specific CD4^+^ T cells, but antigen is transferred from *Mtb*-infected DCs to uninfected bystander DCs in the LNs, for antigen presentation to naive CD4^+^ T cells^22^. On *Mtb* infection, even vaccine-induced memory T cells accumulate in the LNs before mobilization to the lungs^6^. Thus, in the current study, we hypothesized that the delay in accumulation of lung vaccine-induced CD4^+^ T cells in vaccinated *Mtb*-infected hosts is due to a delay in antigen presentation by *Mtb*-infected APCs, thus resulting in a bottleneck and preventing sterilizing immunity to *Mtb* infection. We show that we can overcome the delay in accumulation of vaccine-induced memory CD4^+^ T cells by transferring exogenously primed activated DCs into the lungs of vaccinated mice at the time of *Mtb* infection. DC transfer into vaccinated *Mtb*-infected hosts results in rapid activation of vaccine-induced CD4^+^ T-cell responses, substantial changes in the lung micro-environment, activation of lung alveolar macrophages and early *Mtb* control. Furthermore, these protective mechanisms are dependent on CD103^+^ DCs and the CD40–CD40L activation pathway, as host-directed therapeutics targeting these pathways in vaccinated *Mtb*-infected mice can mimic the protective effects of pulmonary DC transfer. Thus, we have determined a key bottleneck for the failure of TB vaccines to induce sterilizing protection against *Mtb* infection. These results provide a roadmap for the type of immune responses that a sterilizing TB vaccine should induce, representing a milestone in our mechanistic understanding of TB vaccine-mediated immune responses.

## Results

### DC transfer confers superior vaccine-induced Mtb control

**Figure 1:**
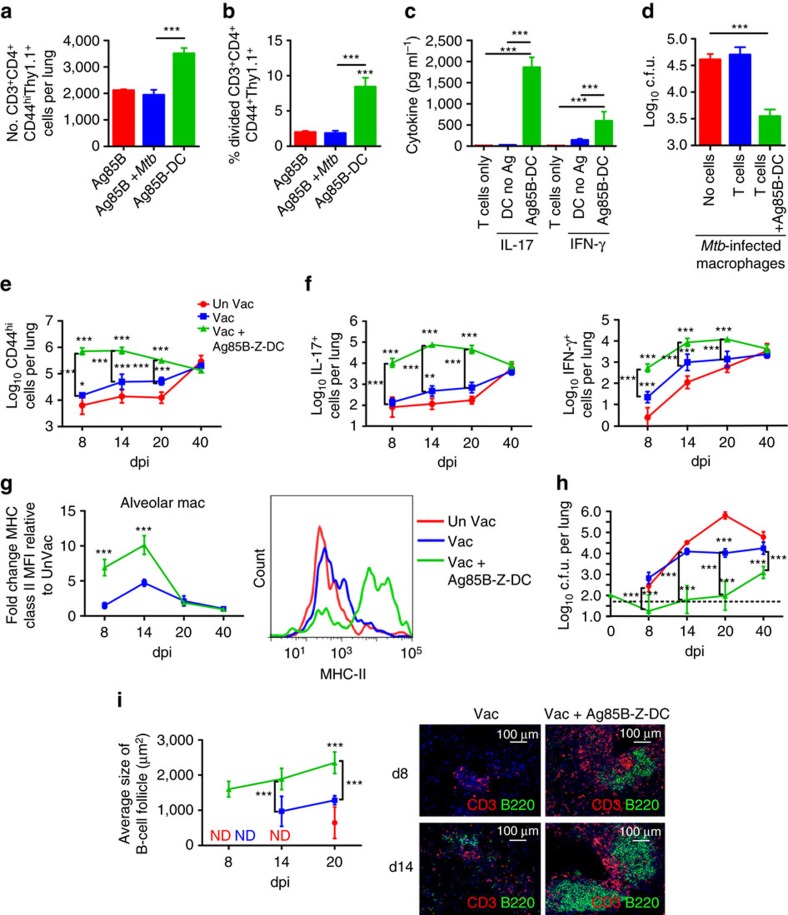
Z-DC transfer in vaccinated mice confers superior *Mtb* control. (**a**–**d**) Naive CD4^+^ T cells from Ag85B TCR Tg Thy1.1^+^ mice were transferred into B6 mice and were vaccinated with BCG s.c. followed by mucosal boost with Ag85B_240–254_ peptide in mucosal adjuvant, rested for 4 weeks, before isolating total lung cells and labelling with CFSE. Cells were cultured for 3 days with either Ag85B antigen alone with or without *Mtb*, or BMDC pulsed with Ag85B antigen. (**a**) Expansion and (**b**) proliferation of *Mtb*-specific cells was assessed by flow cytometry and CFSE dilution. (**c**) Cytokine production was measured in supernatants. However some cytokine levels are not visible on the scale used. (**d**) Six day-stimulated T cells were cultured with *Mtb*-infected BMDMs additional 6 days and intracellular *Mtb* c.f.u. determined by plating. (**e**–**i**) B6 mice were vaccinated with BCG s.c. followed by mucosal boost with Ag85B_240–254_ peptide in mucosal adjuvant, rested for 4 weeks and infected with ∼100 c.f.u. *Mtb* HN878. Vaccinated mice received Ag85B-Z-DC on −1 and 4 dpi. Lungs were collected and flow cytometry was used to assess (**e**) CD44 expression on CD3^+^CD4^+^ T cells, and (**f**) IL-17 and IFN-γ production by Ag85B-specific CD4^+^ CD44^hi^ T cells in the lungs. (**g**) Flow cytometry was used to assess MHC-II mean fluorescence intensity (MFI) on lung alveolar macrophages, and fold change relative to unvaccinated was calculated. A representative plot of MHC-II expression on alveolar macrophages from unvaccinated, vaccinated and vaccinated mice receiving Z-DC transfer is shown at 8 dpi. (**h**) Lung bacterial burden was determined by plating. (**i**) B-cell lymphoid follicle formation was determined by CD3 (red) and B220 (green) staining on formalin-fixed, paraffin-embedded sections by immunofluorescence staining. The average size of B-cell follicles per lobe was quantified using the automated tool of the Zeiss Axioplan 2 microscope in squared microns, representative images of B-cell follicles are shown. (**a**–**d**) *n*=3–4 technical replicates±s.d., (**e**–**i**) *n*=4–5 biological replicates±s.d. *P≤0.05, **P≤0.01, ***P≤0.001 by one-way analysis of variance (ANOVA) (**a**–**d**) or two-way ANOVA (**e**–**i**). ND, not detected. Dotted lines on **h** represent the limit of detection by plating.

Following *Mtb* infection, vaccine-induced CD4^+^ T-cell responses are delayed in vaccinated hosts^3^,^6^, and could be a likely reason for the failure of TB vaccines to induce sterilizing immunity. Therefore, we first assessed whether a delay in accumulation of CD4^+^ T-cell recall responses was due to an inherent deficiency in the ability of the vaccine-induced T cells to respond to antigen exposure. Thus, naive CD4^+^ T cells were isolated from *Mtb*-specific immunodominant antigen 85B (Ag85B) T-cell receptor transgenic (TCR) mice and adoptively transferred into congenic C57BL/6 (B6) mice, which were vaccinated subcutaneously (s.c.) with BCG, rested for 4 weeks followed by a mucosal boost with the CD4^+^ T-cell epitope Ag85B_240–254_. Following a period of rest for 4 weeks, lung CD4^+^ T cells were isolated and cultured *in vitro* in the presence of Ag85B-pulsed DCs. Recall Ag85B-specific CD4^+^ T cells proliferated rapidly, underwent activation, produced cytokines and on co-culture with *Mtb*-infected macrophages could mediate *Mtb* control (Fig. 1a–d). These data suggest that vaccine-induced CD4^+^ T cells can rapidly respond to *Mtb* antigen, but activation is delayed *in vivo* following *Mtb* infection^3^,^6^,^21^. This is not due to a limitation of *Mtb* antigen availability, as infection of vaccinated mice with high doses (∼1,000 c.f.u.) of *Mtb* did not improve vaccine-induced *Mtb* control (Supplementary Fig. 1a)^23^. T regulatory cells induced in response to *Mtb* infection are known to suppress early T-cell responses^24^. As before^25^, however, depletion of T regulatory cells in vaccinated B6 mice early following *Mtb* infection did not improve vaccine-induced *Mtb* control, instead increasing susceptibility to *Mtb* infection (Supplementary Fig. 1b). Thus, although recall CD4^+^ T-cell responses are delayed *in vivo* in *Mtb*-infected hosts^3^,^6^,^21^, vaccine-induced CD4^+^ T cells can rapidly respond *ex vivo* to activate macrophages for *Mtb* control.

To overcome the delayed activation of CD4^+^ T-cell recall responses on *Mtb* infection *in vivo*, we addressed whether delivery of *Mtb* antigen-pulsed DCs into the lungs of *Mtb*-infected-vaccinated mice could overcome this bottleneck and improve recall vaccine-induced CD4^+^ T-cell immunity. B6 mice were thus vaccinated with BCG, rested for 4 weeks, followed by a mucosal boost with Ag85B_240–254_. Mice were further rested for 4 weeks, after which they were challenged with a clinical W-Beijing *Mtb* strain, HN878 (ref. 26). At the time of *Mtb* challenge, vaccinated mice received Ag85B_240–254_-pulsed B6 bone marrow-derived DCs (BMDC) delivered intratracheally (i.t., DC transfer). While vaccination and mucosal boost reduced lung *Mtb* burden as before^4^, B6 DC transfer into vaccinated mice reduced lung *Mtb* burden by ∼98.6%, when compared with the burden in unvaccinated *Mtb*-infected mice (Supplementary Fig. 2a,b). Pulmonary delivery of Ag85B_240–254_ peptide alone did not induce the superior protection seen with DC transfer in vaccinated mice (Supplementary Fig. 2a), further suggesting that antigen availability was not the major reason for delayed vaccine-induced CD4^+^ T-cell recall responses. To further improve the ability of antigen-pulsed DCs to activate CD4^+^ T cells following *Mtb* infection, we next stimulated DCs with the Dectin-1 agonist zymosan (Z-DC)^27^ at the time of antigen pulsing. Activating Z-DC, induced proinflammatory cytokines such as IL-23, IL-1β and IL-6 (Supplementary Fig. 3a–c), but also induced IL-10 (Supplementary Fig. 3d). IL-10 induction can dampen vaccine-induced immunity to *Mtb* infection^28^,^29^, and we next tested whether use of B6 DCs or *Il10*^*−/−*^ DCs treated with zymosan was more effective on transfer at conferring *Mtb* control in vaccinated hosts. B6 DC transfer into vaccinated *Mtb*-infected mice as before reduced *Mtb* burden significantly, when compared with vaccinated *Mtb*-infected hosts, while transfer of B6 Z-DCs had a small but significant improvement in *Mtb* control, when compared with vaccinated hosts receiving B6 DC transfer (Supplementary Fig. 2b). In sharp contrast, we found that vaccinated hosts receiving Z-DCs from *Il10*^*−/−*^ mice resulted in far superior *Mtb* control, with *Mtb* burden-bordering limit of detection by plating assays (Supplementary Fig. 2b, limit of *Mtb* detection is ∼75–100 c.f.u. per lung, *Mtb* burden from three out of five mice were in this range, ∼99.97% reduction in *Mtb* burden, when compared with unvaccinated *Mtb*-infected mice). The near-sterilizing protection in vaccinated mice receiving *Il10*^*−/−*^ Z-DC transfer coincided with increased frequency of both activated IL-17-producing and IFNγ-producing *Mtb*-specific CD4^+^ T cells in the lungs (Supplementary Fig. 2c,d; gating strategy in Supplementary Fig. 4a). Indeed, B6 Z-DC transfer also resulted in increased frequency of lung IL-17-producing *Mtb*-specific CD4^+^ T-cell responses, but failed to improve IFNγ-producing CD4^+^ T-cell responses, when compared with responses in vaccinated hosts (Supplementary Fig. 2c,d). These results together suggest that Z-DCs generated from *Il10*^*−/−*^ mice on transfer into vaccinated hosts can overcome a TB vaccine bottleneck, and confer superior protection against *Mtb* infection by significantly accelerating the accumulation of *Mtb*-specific cytokine-producing CD4^+^ T-cell vaccine responses. Thus, in all experiments in the remainder of the study, Z-DCs from *Il10*^*−/−*^ mice (Ag85B-Z-DC) were used for DC transfer.

### DC transfer accelerates vaccine CD4^+^ T-cell activation

Having established the ability of Z-DC transfer to significantly reduce *Mtb* burden in the lungs of vaccinated mice, we then assessed the kinetics of immune responses in *Mtb* HN878-infected vaccinated mice receiving Z-DC transfer. Vaccine-induced CD4^+^ T-cell responses and activation of lung myeloid cells are not detected in vaccinated hosts until 12–14 dpi following *Mtb* challenge^3^. In contrast, we observed significant early accumulation (8 dpi) of activated CD4^+^ T cells in vaccinated *Mtb*-infected mice receiving Z-DC transfer, when compared with vaccinated *Mtb*-infected mice (Fig. 1e; gating strategy in Supplementary Fig. 4a). In addition, increased numbers of *Mtb*-specific CD44^hi^-activated cells producing the cytokines IL-17 and IFN-γ accumulated early in lungs of vaccinated *Mtb*-infected mice receiving Z-DC transfer, when compared with vaccinated *Mtb*-infected mice or naive *Mtb*-infected mice (Fig. 1f). As noted previously, activated T-cell responses in vaccinated mice not receiving Z-DC transfer accumulated later at 14 dpi (Fig. 1f)^6^, while T-cell responses between the different groups were comparable at later time points. These protective and early activation events in vaccine-induced CD4^+^ T-cell responses coincided with increased early upregulation of major histocompatibility complex (MHC)-II expression on lung alveolar macrophages in Z-DC-recipient-vaccinated *Mtb*-infected mice, when compared with levels expressed in unvaccinated mice (Fig. 1g; gating strategy in Supplementary Fig. 4b). Importantly, the early and rapid activation of vaccine-induced CD4^+^ T-cell responses, and concomitant activation of alveolar macrophages was associated with complete control of *Mtb* growth, maintaining burdens at levels of initial inoculum up to 20 dpi in vaccinated *Mtb*-infected mice receiving Z-DC transfer (Fig. 1h). This is in contrast to naive and vaccinated *Mtb*-infected mice, in which *Mtb* burden increased in the lungs over time (Fig. 1h). Finally, Z-DC transfer at the time of *Mtb* challenge resulted in long-lasting protection up to 40 dpi, with Z-DC transfer-recipient mice still maintaining significantly lower bacterial burdens in the lungs (Fig. 1h). As expected, at later time points, vaccinated mice showed similar *Mtb* burden as unvaccinated mice^30^, suggesting that while Z-DC transfer provides long-lasting vaccine-induced immunity in *Mtb* HN878-infected mice, endogenous vaccine-induced responses are not long-lasting and are lost over time. Furthermore, robust rapid formation of B-cell follicles-harbouring localized T cells could be detected in the lungs of vaccinated *Mtb*-infected mice receiving Z-DC transfer (Fig. 1i), indicating early development of protective lung granulomas. Importantly, even on challenge with a Euro-American *Mtb* strain, H37Rv, we found that Z-DC transfer effectively conferred superior *Mtb* control by activating alveolar macrophages and accelerating accumulation of activated CD4^+^ T cells (Supplementary Fig. 5a–d). Thus, our results suggest that DC transfer can effectively induce superior vaccine control on challenge with different *Mtb* strains. As *Mtb* HN878 is a clinically relevant rapidly emerging *Mtb* strain, all experiments in the remainder of the study was carried out with *Mtb* HN878. Thus, our data together suggest that DC transfer is effective in rapid restimulation of vaccine-induced T cells to produce cytokines and localize within lymphoid follicles to activate macrophages for rapid *Mtb* control, which is sustained long term, and across different *Mtb* strains.

### DC transfer-mediated *Mtb* control is CD4^+^ T-cell dependent

To test the durability of Z-DC transfer in inducing superior vaccine-induced immunity against *Mtb* challenge, BCG-vaccinated mice were boosted mucosally and rested for 10 weeks after vaccination, and Z-DC transfer and *Mtb* HN878 infection were carried out as before. Indeed, we found that Z-DC transfer still induced superior protection in long-term-rested-vaccinated hosts, with *Mtb* burden significantly lower and at levels of detection, when compared with control vaccinated *Mtb*-infected mice that were also rested for 10 weeks before *Mtb* challenge (Supplementary Fig. 6a). Importantly, vaccinated mice receiving Z-DC transfer also showed increased accumulation of activated IL-17^+^ and IFN-γ^+^ CD4^+^ T cells, when compared with CD4^+^ T-cell responses in vaccinated and unvaccinated *Mtb*-infected mice (Supplementary Fig. 6b,c). These data suggest that Z-DC transfer-induced protection confers superior *Mtb* control even following a prolonged period of rest, supporting the durability of DC transfer-mediated protective efficacy in overcoming the TB vaccine bottleneck.

Previous studies have employed administration of DCs as vaccination strategy^31^,^32^, during which mice were immunized with antigen-primed DCs, and then rested before *Mtb* infection. In contrast, the approach taken here is to enhance antigen presentation at the time of *Mtb* infection by administering DCs mucosally at the time of *Mtb* infection. Thus, we tested whether use of Z-DCs as a mucosal boost to BCG vaccination, would similarly induce superior *Mtb* control. Indeed, we show that pulmonary administration of Z-DCs as a mucosal boost to BCG vaccination only had a small protective effect in vaccinated hosts, and did not match the near-sterilizing *Mtb* control seen, when Z-DC transfer was carried out at the time of *Mtb* infection in vaccinated hosts (Supplementary Fig. 6d). These data show that the improved protection conferred by Z-DC transfer is specifically due to the enhanced antigen presentation and accelerated T cells priming at the time of *Mtb* infection, and use of DCs as vaccination tool does not enable the superior *Mtb* control in vaccinated hosts.

**Figure 2:**
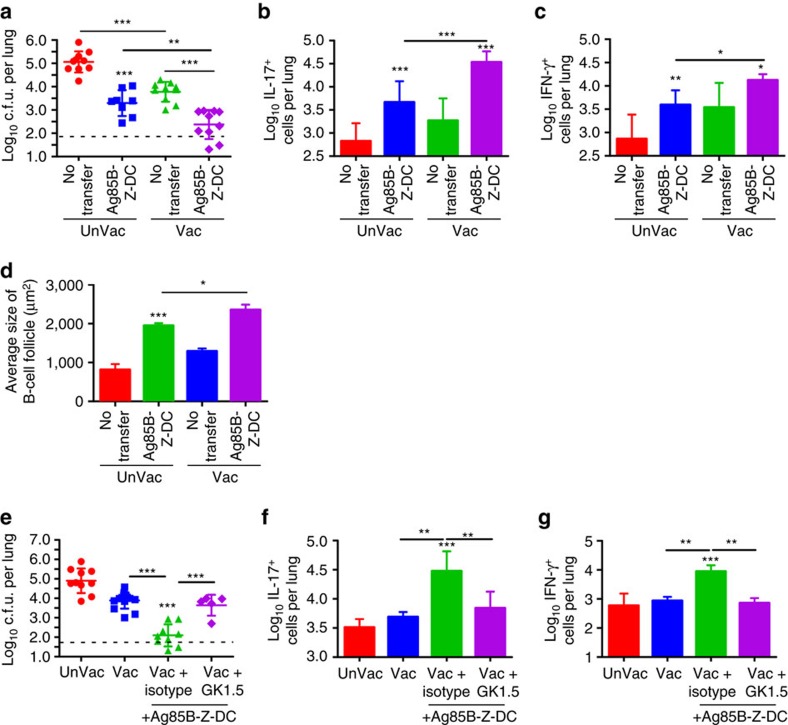
Z-DC transfer-mediated superior *Mtb* control is CD4^+^ T-cell dependent. (**a**–**d**) Unvaccinated B6 mice, or B6 mice vaccinated with BCG s.c. followed by mucosal boost with Ag85B_240–254_ peptide in mucosal adjuvant, were rested for 4 weeks, and *Mtb* HN878-infected (∼100 c.f.u.) and treated with Ag85B-Z-DC on −1 and 4 dpi, and (**a**) bacterial burden was assessed by plating at 20 dpi. Flow cytometry was used to determine production of (**b**) IL-17 and (**c**) IFN-γ by Ag85B-specific CD4^+^CD44^hi^ T cells. (**d**) B-cell lymphoid follicle formation was determined by CD3 (red) and B220 (green) staining on formalin-fixed, paraffin-embedded sections by immunofluorescence staining on 20 dpi. The average size of B-cell follicles per lobe was quantified using the morphometric tool of the Zeiss Axioplan. (**e**–**g**) B6 mice were vaccinated as above, and infected with *Mtb* HN878 (∼100 c.f.u.) via aerosol. Mice received Ag85B-Z-DC transfer on −1 and 4 dpi; and were treated with 300 μg GK1.5 (anti-CD4) or isotype control delivered i.p. on 0 and 7 dpi. Lungs were collected at 20 dpi. (**e**) Lung bacterial burden was determined by plating. Flow cytometry was used to assess (**f**) IL-17 and (**g**) IFN-γ production by Ag85B-specific CD4^+^CD44^hi^ T cells. (**a**–**d**) *n*=8–10 biological replicates±s.d., (**e**–**g**) *n*=5–10 biological replicates±s.d., *P≤0.05, **P≤0.01, ***P≤0.001 by one-way analysis of variance or Kruskall–Wallis test (**e**) or Student's *t*-test (**d**) (UnVac+Ag85B-Z-DC compared with Vac+Ag85B-Z-DC). Dotted lines on bacterial burden plots represent the limit of detection by plating.

Our data show that DC transfer into vaccinated mice at the time of *Mtb* challenge accelerates *Mtb*-specific CD4^+^ T-cell responses and confers superior protection against *Mtb* challenge. Thus, we next addressed whether DC transfer-induced immunity in vaccinated hosts was dependent on vaccine-induced T-cell responses, or whether DC transfer will similarly induce near-sterilizing immunity in previously unvaccinated *Mtb*-infected mice by accelerating priming of naive CD4^+^ T-cell responses. Thus, unvaccinated B6 mice or B6 mice that were BCG vaccinated and mucosally boosted were rested for 4 weeks, and then received Z-DC transfer at the time of *Mtb* challenge, and *Mtb* control was determined. Our data show that while vaccinated *Mtb*-infected mice receiving Z-DC demonstrated superior bacterial control as before, *Mtb* control in unvaccinated mice receiving Z-DC transfer was less effective and resembled *Mtb* control seen in vaccinated hosts not receiving Z-DC transfer (Fig. 2a). Furthermore, although the numbers of IL-17 and IFN-γ-producing *Mtb*-specific CD4^+^ T cells in Z-DC transfer-recipient *Mtb*-infected mice were significantly higher, when compared with *Mtb*-infected mice not receiving Z-DC, the numbers were reduced, when compared with numbers seen in vaccinated mice receiving Z-DC transfer (Fig. 2b,c). In addition, T-cell localization within B-cell follicles in vaccinated mice receiving Z-DC transfer was higher, when compared with unvaccinated mice that received Z-DC transfer (Fig. 2d). These results together suggest that DC transfer into unvaccinated hosts can also accelerate the priming of naive CD4^+^ T cells into cytokine-producing *Mtb*-specific CD4^+^ T-cell responses and improve *Mtb* control. However, the protection in naive *Mtb*-infected mice receiving Z-DC transfer is not as effective as seen in vaccinated mice receiving Z-DC transfer, suggesting that Z-DC transfer mediating near-sterilizing immunity occurs only in previously vaccinated hosts.

To further confirm that DC transfer-mediated protection is vaccine-induced CD4^+^ T-cell-dependent, vaccinated mice receiving Z-DC transfer received a CD4 depleting antibody at early time points following *Mtb* challenge, and *Mtb* control in vaccinated hosts receiving Z-DC transfer was assessed at 20 dpi. Our data show that early depletion of CD4^+^ T cells in vaccinated mice receiving DC transfer reversed the superior protection (Fig. 2e) and coincided with loss of early accumulation of IFN-γ- and IL-17-producing *Mtb*-specific CD4^+^ T-cell responses (Fig. 2f,g). These results together project that the near-sterilizing protection seen on DC transfer in vaccinated host is dependent on the presence of vaccine-induced CD4^+^ T-cell responses, as either absence of vaccination or absence of CD4^+^ T cells abrogates the superior near-sterilizing protection.

### DC transfer overcomes bottlenecks in different vaccinations

**Figure 3:**
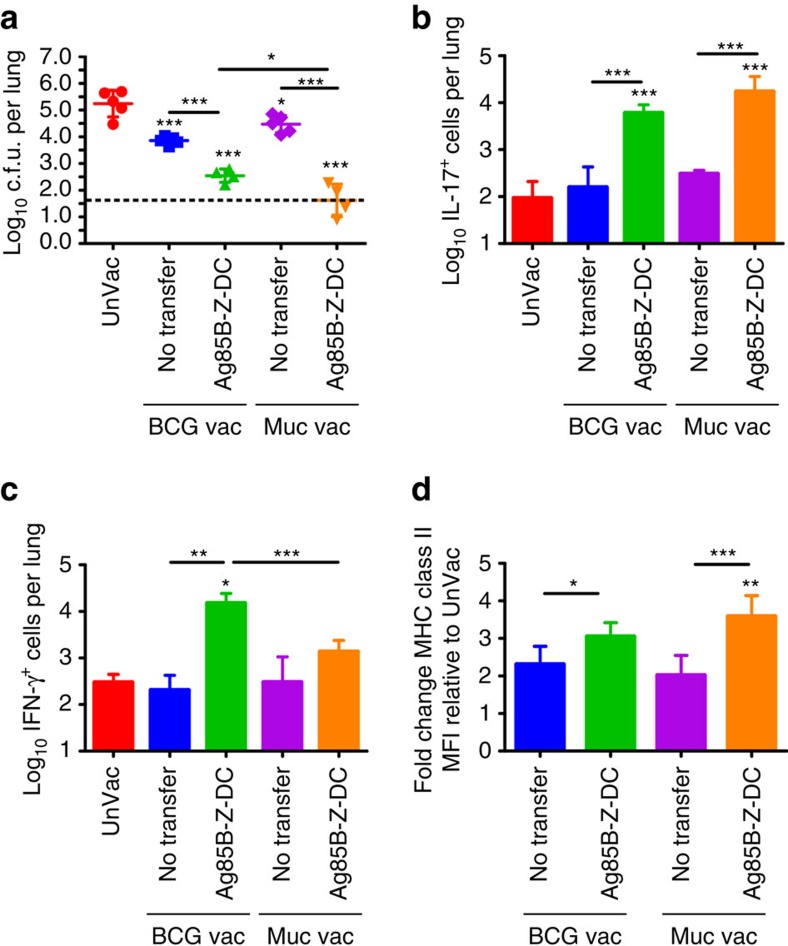
Z-DC transfer improves *Mtb* control in different vaccines. Mice were mucosally vaccinated with three doses of Ag85B_240–254_ peptide in mucosal adjuvant, or parenterally vaccinated with BCG s.c., rested for 4 weeks, and were *Mtb* HN878-infected (∼100 c.f.u.) and received Ag85B-Z-DC transfer as described previously. (**a**) Lung bacterial burden was assessed at 20 dpi by plating. Flow cytometry was used to determine (**b**) IL-17 and (**c**) IFN-γ production by Ag85B-specific CD4^+^ CD44^hi^ T cells. (**d**) The mean fluorescence intensity (MFI) was calculated to determine level of MHC-II expression on alveolar macrophages, and fold change MFI relative to UnVac was calculated for each group at 20 dpi. *n*=4–5 biological replicates±s.d. *P≤0.05, **P≤0.01, ***P≤0.001 by one-way analysis of variance or Kruskall–Wallis test (**c**), or Student's *t*-test (**d**) (BCG Vac compared with BCG Vac+Ag85B-Z-DC). Dotted lines on bacterial burden plots represent the limit of detection by plating.

Our data show that Z-DC transfer accelerates vaccine-induced *Mtb*-specific CD4^+^ T-cell responses in mice vaccinated with BCG and mucosally boosted with Ag85B_240–254_ to confer superior protection on *Mtb* challenge. Thus, we next tested whether Z-DC transfer could be broadly used to provide superior protection in different vaccination strategies. Recent work by us and others has highlighted the importance of a lung-resident T-cell responses following mucosal vaccination in protection against TB disease^4^,^5^. In addition, mucosal vaccination induces potent lung-resident CD4^+^ T-cell responses, while standard parenteral BCG vaccination induces potent systemic vaccine-induced CD4^+^ T-cell responses (Supplementary Fig. 7a,b). Thus, we assessed whether Z-DC transfer would improve *Mtb* protection when used in standard BCG vaccination, when compared with mice that only received mucosal vaccination. B6 mice were vaccinated with either a mucosal TB vaccine consisting of Ag85B_240–254_ in mucosal adjuvant^4^ or parenterally with BCG^28^, rested, and on *Mtb* challenge received Z-DC transfer. Z-DC transfer provided superior vaccine-induced *Mtb* control in both mucosally vaccinated and BCG-vaccinated mice (Fig. 3a), suggesting that DC transfer functions broadly in different vaccination strategies. We found that while Z-DC transfer in mucosally vaccinated mice induced accumulation of large numbers of IL-17-producing *Mtb*-specific CD4^+^ T-cell responses, IFN-γ-producing CD4^+^ T-cell responses were not significantly increased (Fig. 3b,c). In contrast, BCG-vaccinated mice receiving DC transfer exhibited increased both IFN-γ and IL-17 CD4^+^ T-cell responses (Fig. 3b,c). Indeed, the improved CD4^+^ T-cell recall responses in vaccinated hosts receiving Z-DC transfer coincided with enhanced activation of alveolar macrophages when compared to unvaccinated hosts (Fig. 3d). Thus, our data show that although the effector cytokine mechanism by which Z-DC transfer confers superior protection in vaccination models may be different, Z-DC transfer can rapidly activate both systemic and mucosal CD4^+^ T-cell responses to induce superior protection against *Mtb* control in different vaccine strategies and is broadly applicable.

### Identifying host pathways to overcome TB bottlenecks

**Figure 4:**
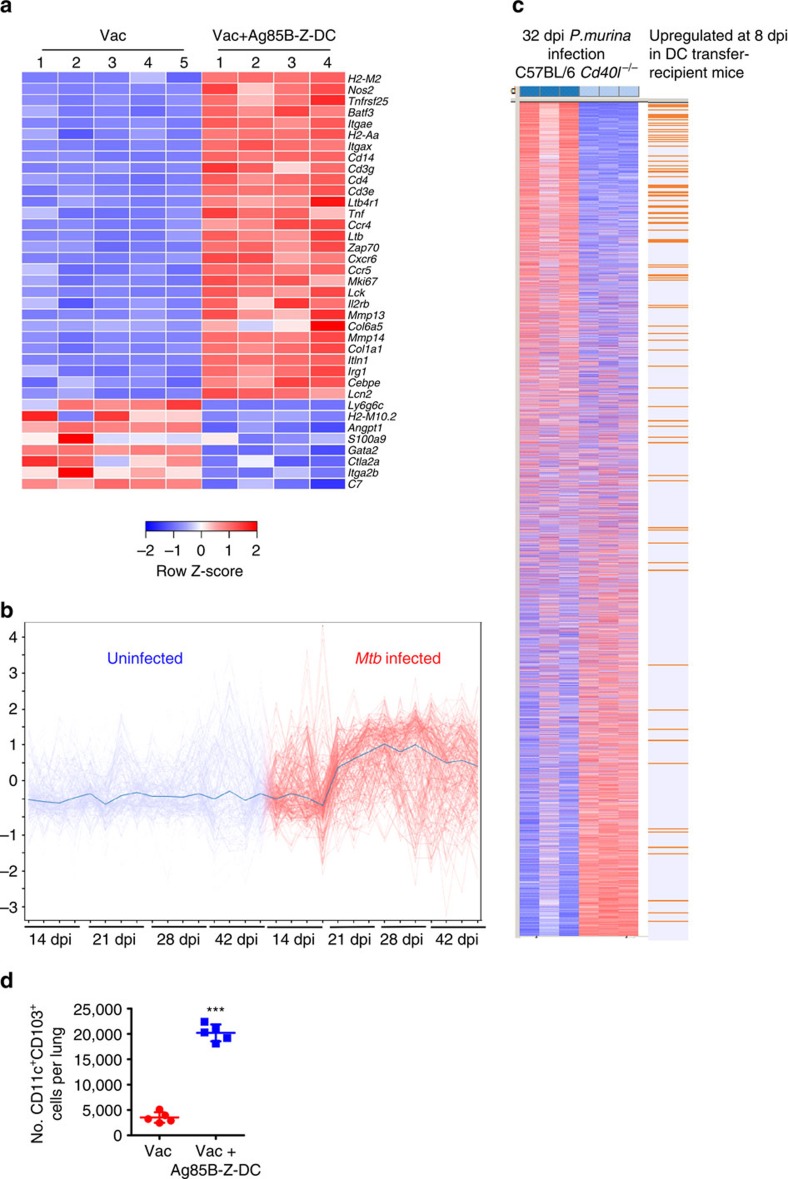
Z-DC transfer induces early genes associated with APC activation. B6 mice were vaccinated with BCG s.c. followed by mucosal boost with Ag85B_240–254_ peptide in mucosal adjuvant, rested for 4 weeks, and *Mtb* HN878-infected (∼100 c.f.u.) and treated with Ag85B-Z-DC on −1 and 4 dpi. RNA-seq was performed on lungs at 8 dpi. (**a**) Expression values of each biological replicate (*n*=5 for Vac, *n*=4 for Vac+Ag85B-Z-DC) for each group of a subset of selected genes of interest (all selected genes had a differential expression of at least twofold). These genes were differentially expressed using CuffDiff output with a *P*<0.05, and Benjamini–Hochberg false discovery rate (FDR) of 5%. The scaled expression of each replicate, denoted as the row *Z*-score, is plotted in red–blue colour scale with red indicating high expression and blue indicating low expression. *q* values for each gene of interest are shown in Supplementary Table 1 for the top 100 upregulated genes. (**b**) Expression of the top 100 upregulated genes shown in Supplementary Table 1 was considered in the published transcriptional data following expression in lungs of mice infected (red curves) with *Mtb,* or uninfected controls (blue curves) along the timecourse of 14, 21, 28 and 42 dpi^33^. (**c**) Gene Set Enrichment Analysis was used to analyse the expression of the top 100 upregulated genes shown in Supplementary Table 1 in the context of the infection model of *Cd40l*^*−/−*^ mice with *P. murina*. Genes differentially expressed between wild type (WT) and *Cd40l*^*−/−*^ mice on 32 days post-*P. murina* infection were compared and the top 1,000 expressed genes shown in the heat map, ranked by the level of differential expression between the two genotypes^70^. Orange lines indicate positions of 100 genes from Supplementary Table 1, *P*<10^−3^. (**d**) Numbers of CD11c^+^CD103^+^ cells in lungs of *Mtb*-infected-vaccinated Z-DC-recipient mice was determined using flow cytometry. *n*=4–5 biological replicates±s.d. ***P≤0.001 by Student's *t*-test.

Transfer of exogenously primed DCs into vaccinated mice can effectively accelerate the vaccine-induced CD4^+^ T-cell response following *Mtb* infection, and improve bacterial control. DC transfer as therapy for use in *Mtb*-infected individuals in TB-endemic areas; however, is logistically difficult to translate for human use as timing of *Mtb* exposure is impossible to predict. Thus, to identify endogenous host pathways that could be targeted for future use, we carried out transcriptional profiling in lungs of vaccinated *Mtb*-infected mice receiving DC transfer, and compared it with gene expression profiles in lungs from *Mtb*-infected-vaccinated mice. Several genes involved in early T-cell activation, macrophage function, as well as chemokines involved in B-cell follicle formation, were significantly upregulated (Supplementary Tables 1 and 2; Fig. 4a; validated in Supplementary Fig. 8). We found that the genes induced rapidly at 8 dpi in vaccinated *Mtb*-infected mice receiving Z-DC transfer, were not upregulated until 21 dpi in *Mtb*-infected mice (Fig. 4b)^33^. These results further support our findings that protective immune responses are substantially accelerated in vaccinated *Mtb*-infected mice receiving Z-DC transfer.

To identify pathways that were being induced in lungs of vaccinated mice receiving Z-DC transfer, we cross referenced the transcriptional signature of the vaccinated Z-DC-recipient *Mtb*-infected mice (Supplementary Table 1) against all publicly available RNASeq datasets. In this unbiased search, we found the significant regulation of immune pathways associated with early T-cell activation and APC function, present in our transcriptional data also coincided with expression in the lungs of wild-type mice infected with the pulmonary pathogen, *Pneumocystis murina*. Specifically, we found that several genes were induced both in the lungs of vaccinated *Mtb*-infected mice receiving Z-DC transfer, and following *P. murina* infection in wild-type mice. In contrast, these genes were not upregulated in *Cd40l*^*−/−*^ mice infected with *P. murina* (Fig. 4c), suggesting CD40 axis dependence underlying the accelerated vaccine response in Z-DC transfer-recipient mice. Furthermore, Batf3 is a transcription factor that drives development of CD103^+^ DCs, which are important for priming Th1 and Th17 responses^34^,^35^. *Batf3* messenger RNA expression was increased in vaccinated *Mtb*-infected hosts receiving Z-DC transfer (Supplementary Fig. 8) and coincided with increased accumulation of Batf3-dependent CD103^+^ DCs (Fig. 4d). Thus, we have identified a CD103^+^ DC and CD40 pathway that can potentially be targeted for improved *Mtb* control in vaccinated mice.

### Activating CD103^+^ DCs improves *Mtb* control by vaccines

**Figure 5:**
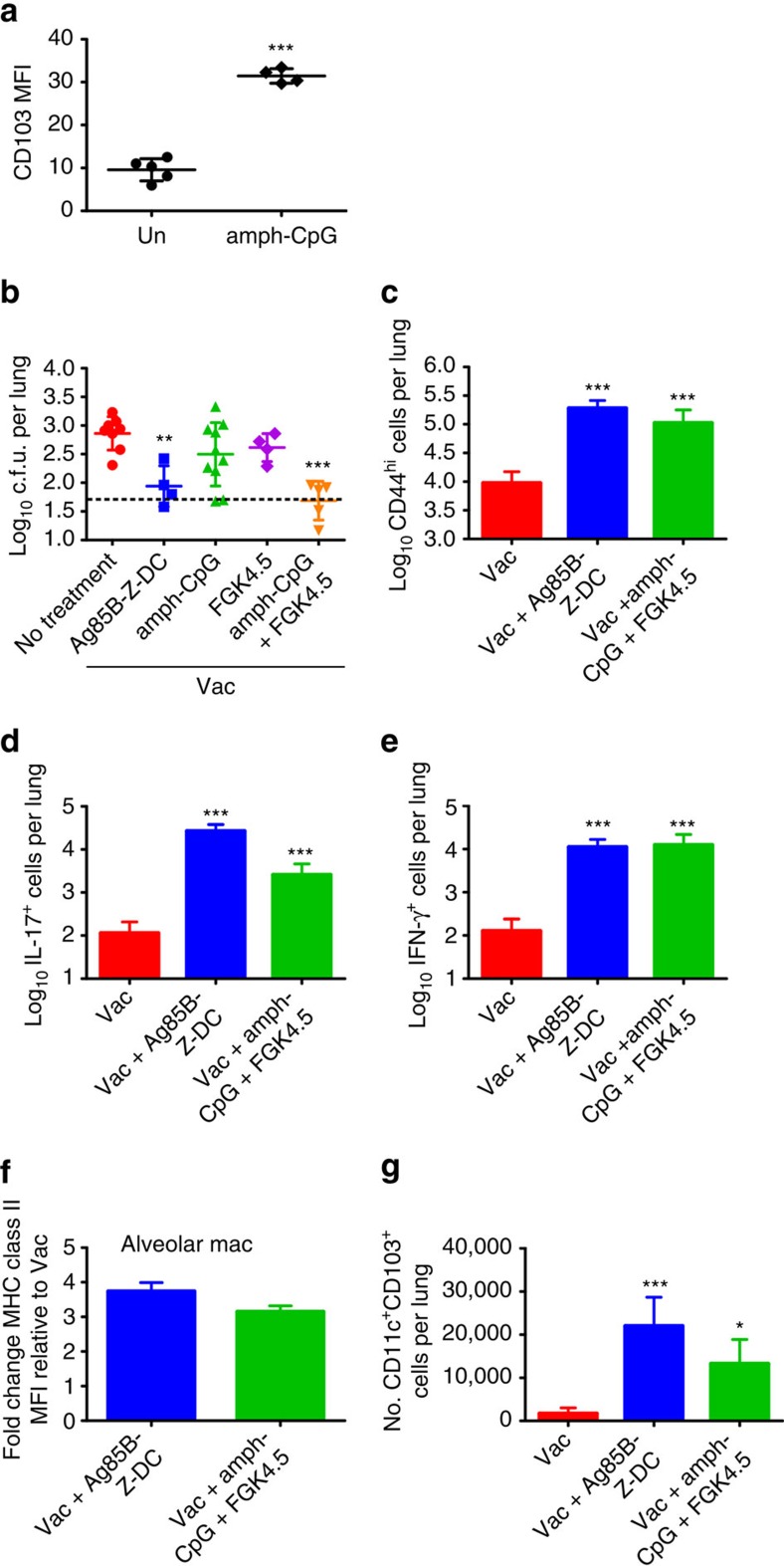
Amph-CpG and CD40 agonist improves *Mtb* control in vaccinated hosts. (**a**) BMDC were cultured from B6 mice. After harvesting and resting overnight, BMDC were treated overnight with 1.24 nmol amph-CpG. CD103 expression on activated DCs was assessed by flow cytometry. (**b**–**g**) Mice were vaccinated with BCG s.c. and rested for 4 weeks. Vaccinated mice were infected with *Mtb* HN878 and treated with either Ag85B-Z-DC, amph-CpG (1.24 nmol), FGK4.5 (100 μg), or both amph-CpG and FGK4.5 along with Ag85B peptide (5 μg) on −1 and 4 dpi. Mice were collected at 8 dpi. (**b**) Lung bacterial burden was determined by plating. Flow cytometry was used to assess (**c**) numbers of CD4^+^CD44^hi^ cells in the lungs, and (**d**) IL-17 and (**e**) IFN-γ production by CD4^+^ T cells, (**f**) MHC-II expression on alveolar macrophages and fold change MFI relative to Vac was calculated. (**g**) Flow cytometry was used to calculate numbers of CD11c^+^CD103^+^ cells in the lungs. (a) *n*=4–5 technical replicates±s.d., (**b**–**g**) *n*=4–10 biological replicates. *P≤0.05, **P≤0.01, ***P≤0.001 by Student's *t*-test (**a**) or one-way analysis of variance (**b**–**g**). Dotted lines on bacterial burden plots represent the limit of detection by plating.

CD103^+^ DCs accumulate in vaccinated *Mtb*-infected hosts receiving DC transfer, and the CD40 pathway is activated in Z-DC transfer-induced protection against TB disease in vaccinated hosts. To target endogenous host mucosal DCs, we then assessed if activation of the CD40 pathway and targeting endogenous CD103^+^ mucosal DCs in standard BCG-vaccinated *Mtb*-infected mice, can mimic the effects of DC transfer and induce superior *Mtb* control. Amphiphilic-CpG (amph-CpG) in mice is a modified Toll-like receptor (TLR) 9 agonist that is efficiently taken up by DCs and macrophages, and enhances T-cell responses to peptide vaccines^36^. In addition, treatment of DCs with amph-CpG upregulates CD103 expression (Fig. 5a). FGK4.5 is an agonistic CD40 antibody that has been shown to activate DCs in murine cancer treatment models^37^,^38^. Thus, vaccinated *Mtb*-infected mice received amph-CpG along with FGK4.5, or either amph-CpG or FGK4.5 alone, delivered i.t. at −1 and 4 dpi, and early vaccine responses and *Mtb* burden in the lungs were assessed. We found that while delivery of either amph-CpG alone or FGK4.5 alone did not induce significant vaccine protection, delivery of amph-CpG and FGK4.5 together in vaccinated mice results in early *Mtb* control, similar to that seen in vaccinated *Mtb*-infected hosts receiving DC transfer (Fig. 5b). Importantly, delivery of amph-CpG and FGK4.5 induced accelerated activation of vaccine-induced cytokine-producing CD4^+^ T-cell responses (Fig. 5c–e) and coincided with activation of lung alveolar macrophages (Fig. 5f). Moreover, treatment with amph-CpG and FGK4.5 resulted in increased accumulation of CD103^+^ lung DCs in *Mtb*-infected-vaccinated mice (Fig. 5g; see gating strategy in Supplementary Fig. 4c). Thus, our data suggest that targeting host endogenous CD103^+^ DCs and activation of the CD40 pathway can overcome delayed antigen presentation and results in rapid activation of vaccine-induced T-cell responses and completely control *Mtb* growth.

## Discussion

Thus far, the development of sterilizing vaccines against TB for human use has not become a reality. This is especially concerning considering the status of TB, as one of the leading causes of death due to infectious diseases in the world today. In the current study, we hypothesized that our inability to develop a sterilizing vaccine against *Mtb* infection is not due to a failure of the vaccine to induce effective T-cell responses, but due to a delay in antigen presentation and subsequent delay in accumulation of vaccine-induced T-cell responses. Indeed, we show here that vaccine-induced T cells can respond to antigen exposure and induce mycobactericidal activity *ex vivo*. Transfer of exogenously primed DCs into lungs of vaccinated *Mtb*-infected mice can directly activate vaccine-induced T cells *in vivo,* leading to accelerated formation of protective B-cell follicles, rapid and early alveolar macrophage activation, and early control of establishment of *Mtb* infection. In addition, our results have identified novel mechanisms, involving CD103^+^ DCs and the CD40 activation pathway that may be therapeutically targeted to improve vaccine-induced protection during TB. Together, our data provide novel and mechanistic insights into pathways that can be targeted to generate sterilizing vaccine-induced immunity against TB.

Most modern TB vaccines induce lung and systemic Th1 and/or Th17 vaccine responses^2^. In animal models, however, none of these vaccines induce sterilizing immunity to TB, instead only decreasing the lung *Mtb* burden by 0.5–1.0 log (refs 3, 8, 9, 10, 11). Our studies show that vaccine-induced CD4^+^ *Mtb*-specific T cells isolated from the lung can undergo activation rapidly, proliferate and produce both IFN-γ and IL-17 *ex vivo*. Importantly, we show that proliferating vaccine-induced CD4^+^ T cells can activate macrophages to control *Mtb*, suggesting that there is no inherent deficiency in vaccine-induced CD4^+^ recall responses. In addition, lung-resident vaccine-induced CD4^+^ T-cell responses are long lasting^39^ and adoptive transfer of T cells isolated from *Mtb*-infected animals into newly infected animals confers protective immunity^40^,^41^,^42^,^43^,^44^. *Mtb* is a successful pathogen, known to inhibit MHC-II transactivator expression, MHC-II expression and antigen presentation in APCs^18^. Thus, the inability of TB vaccines to confer sterilizing immunity on *Mtb* challenge may be associated with delayed CD4^+^ T-cell responses due to delayed APC function, rather than just poor induction of vaccine-induced CD4^+^ T cells. Accordingly, targeting DCs in vaccinated hosts can substantially accelerate the activation and accumulation of cytokine-producing CD4^+^ T-cell recall responses in the lung, initiate rapid activation of alveolar macrophages and limit *Mtb* growth. Interestingly, pre-clinical vaccines that have come closest to inducing sterilizing immunity following *Mtb* infection have been recombinant mycobacterial vaccines delivered mucosally^15^,^45^. BCG is known to reduce the overall childhood morbidity caused by infections other than *Mtb*^46^, which could be due to the ability of BCG to induce ‘innate memory' by epigenetically altering the phenotype of infected cells to one producing increased inflammatory cytokines, via autophagy and nucleotide-binding oligomerization domain-containing protein 2 (NOD2)-dependent pathways^47^,^48^. Thus, it is possible that recombinant mycobacterial vaccines delivered mucosally target pathways similar to DC transfer through activation of lung-resident DCs and macrophages, thereby driving earlier activation of lung-resident T cells on *Mtb* challenge. Further work delineating the specific mechanisms by which vaccine-induced immunity can be accelerated by targeting these pathways will therefore be highly relevant.

Previous studies have attempted to improve DC function at the time of vaccination by targeting the CD40 activation pathway^49^, DC receptor Dec-205 (refs 50, 51), or by utilizing cell-derived vaccines, such as *Mtb* antigen-primed DCs^31^,^32^. Improving DC function by targeting the CD40 pathway during vaccination had no effect^49^, while activating Dec-205 at the time of vaccination had a small effect (∼0.5 log reduction) on vaccine-induced protection on *Mtb* challenge^50^,^51^. Vaccination with antigen-primed DCs has generated mixed results in *Mtb*-infected mice, with a study demonstrating a negative impact of DC vaccination due to exuberant inflammation^31^, or induction of vaccine-induced protection similar to levels seen in BCG-vaccinated hosts^32^. Our results show that use of Z-DCs as a mucosal vaccination tool as boost to BCG vaccination has a small protective effect on *Mtb* infection, but not as effective as use of Z-DC transfer at the time of infection. In contrast, only very few studies have therapeutically manipulated early innate responses following *Mtb* infection. Delivery of polyI:C, a TLR3 ligand, exacerbated inflammation and increased *Mtb* burden^52^. In contrast, delivery of FimH, a TLR4 ligand, improved T-cell responses and resulted in ∼1 log reduction in lung bacterial burden, following high-dose intranasal infection with *Mtb* H37Ra (ref. 53). To our knowledge, no published studies have manipulated early host responses in vaccinated *Mtb*-infected hosts, with resulting complete early *Mtb* control. Furthermore, both CpG (reviewed in refs 54, 55) and CD40 agonists (reviewed in ref. 56) have been safely tested in clinical human trials, predominantly for cancer treatment. Thus, repurposing these host-directed therapeutics, as well as other activators of CD103^+^ DC function^57^,^58^, for rapid activation of vaccine-induced T-cell responses may thus be useful in development of novel drugs or vaccination approaches for TB.

Our results show that while delivery of unstimulated DCs by itself improves vaccine-induced CD4^+^ T-cell responses and *Mtb* control in vaccinated hosts, zymosan activation of DCs, especially generated from Il10^−/−^ mice confers superior vaccine-induced *Mtb* control. These results support the idea that delayed antigen presentation and subsequent delayed induction of vaccine-induced T-cell activation is a major bottleneck for TB vaccine efficacy. In addition, these results support an important role for IL-10 in limiting TB vaccine efficacy and early *Mtb* control in vaccinated hosts^28^,^29^. Furthermore, Z-DC transfer works in both standard BCG vaccinated as well as in mucosally vaccinated *Mtb*-infected hosts, the route of vaccination may define the mechanisms of protection. In mucosally vaccinated *Mtb*-infected mice receiving DC transfer, mucosal lung-resident CD4^+^ T cells, primarily IL-17-producing cells are activated and accumulate rapidly in the lung, thus mediating the superior protection associated with this model. In BCG-vaccinated *Mtb*-infected mice receiving DC transfer, DC transfer can still prime a population of lung-resident IL-17-producing vaccine-induced CD4^+^ T cells, but likely DC migration to LNs is required to mobilize IFN-γ-producing vaccine-induced CD4^+^ T-cell responses from the systemic pool, to provide enhanced *Mtb* control. Regardless, that we can target CD103^+^ DCs and the CD40 activation pathway to limit *Mtb* growth in standard BCG-vaccinated *Mtb*-infected hosts suggest that our strategy is effective in overcoming a bottleneck associated with delayed induction of vaccine-induced CD4^+^ T-cell responses in vaccinated hosts. This is further supported by our results showing that DC transfer can protect vaccinated hosts on challenge with *Mtb* strains belonging to different lineages, suggesting that T-cell bottleneck for TB vaccines is universal. In addition, that Z-DC transfer into unvaccinated hosts can also accelerate naive T-cell priming and improve primary immunity, albeit not to the effectiveness seen in vaccinated hosts, suggest the DC transfer may be useful to enhance both primary and vaccine-induced pathways during *Mtb* infection.

DC transfer not only induces the rapid activation of vaccine-induced CD4^+^ T-cell responses in vaccinated *Mtb*-infected mice, but also has substantial downstream effects on activation of myeloid pathways in the lung. First, lung alveolar macrophages are rapidly activated in vaccinated hosts by 8 dpi, an event that does not happen until 14 dpi in vaccinated hosts and until 21 dpi in unvaccinated hosts^3^. Accordingly, the early global transcriptional changes in the lungs of vaccinated *Mtb*-infected mice receiving DC transfer mirror the transcriptional changes occurring in 21 dpi *Mtb*-infected lungs^33^. Furthermore, we observe early accumulation of myeloid DCs, including CD103^+^ DCs and an enhancement of DC activation pathways, in particular genes associated with CD40 activation in the lungs of vaccinated *Mtb*-infected mice. CD103^+^ DCs are a tissue-resident DC subset, which have primarily been implicated in cross-presentation and induction of CD8^+^ T-cell responses^59^,^60^. More recently, however, CD103^+^ DCs have been shown to induce CD4^+^ T cells^34^,^35^,^61^. In the lung, CD103^+^ DCs represent a migratory DC subset, with the ability to migrate from the lungs to the LN and back again^62^, and induce both Th1 and Th17 cytokines by CD4^+^ T cells^34^,^35^,^63^. Although antigen presentation by transferred DCs likely directly activates vaccine-induced CD4^+^ T-cell responses, rapid activation of CD4^+^ T-cell responses also modifies the lung micro-environment to improve subsequent antigen presentation by endogenous host APCs. We show here that targeting DC activation pathways through the TLR ligand amph-CpG, along with use of the CD40 agonist FGK4.5, similarly control *Mtb* growth. Treatment of DCs with granulocyte–macrophage-colony-stimulating factor (GM-CSF), a by-product of TLR9 stimulation through CpG^64^ has previously been shown to upregulate CD103 expression^65^. Thus, it is possible that CpG stimulates CD103^+^ expression on DCs indirectly through inducing GM-CSF by other lung-resident cells. Upregulation of CD103 as well as recruitment of CD103^+^ DCs to the lung alone, however, are not sufficient for improved T-cell activation, suggesting that the activating signal delivered through the CD40 agonist FGK4.5 provides the second signal required for improved early antigen presentation to vaccine-induced CD4^+^ T cells. Therefore, *Mtb-*infected individuals likely have a population of antigen-responsive T cells, and targeting DCs as a potential therapy, may provide the lung-resident T cells the necessary signals to proliferate and control *Mtb* infection.

Thus, our data for the first time show that delayed activation of vaccine-induced T-cell responses is a critical bottleneck for TB vaccine efficacy. Our results demonstrate that this delay can be overcome by DC transfer, or by targeting DC activation, leading to rapid T-cell recall responses and enhanced alveolar macrophage activation, resulting in complete control of early *Mtb* growth. These results provide a roadmap for the type of immune responses that a sterilizing TB vaccine should induce, representing an important milestone in our mechanistic understanding of TB vaccine-mediated immune responses. Our results suggest that despite induction of effective *Mtb*-specific vaccine T-cell responses by modern TB vaccines^66^,^67^,^68^, delayed T-cell vaccine responses on *Mtb* infection may pose a significant hurdle to development of a sterilizing vaccine for TB in humans. Therefore, it is possible that the development of a sterilizing vaccine for TB may not be a realistic goal. Instead, redirecting our efforts towards developing novel vaccine strategies and therapeutics for preventing the reactivation of latent TB and TB transmission may benefit global control of TB.

## Methods

### Mice

C57BL/6 (B6), B6.129P2-*Il10*^*tm1Cgn*^/J (*Il10*^*−/−*^) and B6.129 (Cg)-*Foxp3*^*tm3(DTR/GFP)Ayr*^/J (Foxp3.DTR) mice were purchased from Jackson Laboratories (Bar Harbor, ME, USA) and bred at Washington University in St Louis. C57BL/6-Tg(H2-Kb-Tcra,-Tcrb)P25Ktk/J (P25 TCR Tg) and B6.PL-*Thy1*^*a*^/CyJ (Thy1.1) mice were purchased from Jackson Laboratories, and crossed and bred at Washington University in St Louis. Mice (male and female) were used at 6–8 weeks of age. Experiments were designed according to empirical statistical power analysis. All mice were used and housed in accordance with the National Institute of Health guidelines for housing and care of laboratory animals, and permission for the experiments in this study was granted by the Washington University in St Louis Institutional Animal Care and Use Committee under protocol 20130195. All efforts were made to minimize pain and suffering as described in this protocol.

### Bacterial infection and vaccination

*M. bovis* Bacille Calmette–Guerin (BCG Pasteur, Source: Trudeau institute), *Mtb* strain HN878 (Source: BEI Resources) and *Mtb* strain H37Rv (Source: Trudeau Institute) were grown to mid-log phase in Proskauer Beck medium containing 0.05% Tween80 and frozen in 1 ml aliquots at −80 °C.

Mice were vaccinated with 1 × 10^6^ c.f.u. BCG s.c.^4^ and 4 weeks later received 133 μg Ag85B_240–254_ peptide (New England Peptide, Gardner, MA, USA) along with 1 μg heat-labile enterotoxin (LT-IIb) intranasally in 20 μl (10 μl per nare). In some experiments, mice only received mucosal vaccination with three doses, 2 weeks apart of 133 μg Ag85B_240–254_ peptide in 1 μg LT-IIb intranasally. In other experiments, mice only received 1 × 10^6^ c.f.u. BCG s.c. as a model of parenteral vaccination. Four or 10 weeks after the final vaccination, mice were infected with 100 c.f.u. (low dose) or 1,000 c.f.u. (high dose) *Mtb* HN878 via aerosol using a Glas-Col aerosol exposure system (Glas-Col, Terre Haute, IN, USA)^4^. At given time points following infection, organs were collected, homogenized and tissue homogenates plated in serial dilutions on 7H11 agar (BD Biosciences, San Jose, CA, USA) to assess bacterial burden^4^.

In experiments using Foxp3.DTR mice, mice were treated with three doses 2 days apart of 10 μg kg^−1^ diphtheria toxin (Millipore, Billerica, MA, USA)^69^ for depletion of Foxp3^+^ cells, starting 8 dpi.

### *In vitro* culture of DCs and macrophages

BMDC and bone marrow-derived macrophages (BMDMs) were generated from B6 or *Il10*^*−/−*^ mice. Cells were isolated from the femur and tibia, and cultured at 1 × 10^6^ cells ml^−1^ in 10 ml complete DMEM (cDMEM) supplemented with 20 ng ml^−1^ recombinant mouse GM-CSF (Peprotech, Rocky Hill, NJ, USA) for 3 days at 37 °C in 7.5% CO_2_, at which point, an additional 10 ml cDMEM supplemented with 20 ng ml^−1^ recombinant mouse GM-CSF was added. Non-adherent cells (BMDCs) were collected on the seventh day of culture, and counted and plated at 2 × 10^6^ cells ml^−1^ in cDMEM. Adherent BMDMs were collected at the same time by scraping and cultured at 1 × 10^6^ cells ml^−1^. For BMDC stimulation, Ag85B_240–254_ peptide (20 μg ml^−1^) and Zymosan (Invivogen, San Diego, CA) was added at 25 μg ml^−1^. Cells were stimulated for 16 h before being collected, washed, counted and instilled i.t. into mice at 1 × 10^6^ cells in 50 μl on the day before infection and 4 dpi. Supernatants from stimulated *in vitro* cultures were collected and frozen at −80 °C for analysis by enzyme-linked immunosorbent assay and multiplex assay. In some experiments, BMDCs were treated with 1.24 nmol amph-CpG.

### Amph-CpG and FGK4.5 treatment of mice

Amph-CpG was prepared as described previously^36^. Briefly, solid phase DNA synthesis and 5′ lipophilic conjugation were carried out using an ABI 394 synthesizer^36^. The sequence used was murine oligodeoxynucleotides (ODN) class B sequence 1,826 with two guanine spacers: 5′-diacyl lipid-*G*G*T*C*C*A*T*G*A*C*G*T*T*C*C*T*G*A*C*G*T*T-3′. Amph-CpG was delivered to mice in 50 μl i.t. at 1.24 nmol per mouse. The CD40 agonist FGK4.5 (R&D Systems, Minneapolis, MN, USA) was delivered in 50 μl i.t. at 100 μg per mouse. Both treatments were delivered with 5 μg Ag85B peptide.

### Antibody treatment of mice

In some experiments, mice were treated with CD4 (clone GK1.5, BioXcell) depleting antibodies delivered intraperitoneally, starting on the day of *Mtb* infection and 7 days later (300 μg per mouse).

### Generation of single-cell suspensions from tissues

Lung single-cell suspensions from vaccinated or *Mtb*-infected mice were generated as before^4^. For LNs and spleens, organs were passed through a 70 μm cell strainer and then processed as for lungs^4^. For flow cytometric analysis, cells were either stained immediately, or stimulated for 18 h in the presence of Ag85B_240–254_ peptide, with GolgiStop (5 μl ml^−1^; BD Biosciences) and Brefeldin A (1 μg ml^−1^; BioLegend, San Diego, CA, USA) added for the last 5 h. Intracellular cytokine staining was performed using the BD Cytofix/Cytoperm kit (BD Biosciences). Cells were collected using a BD FACS Jazz or a BD LSR Fortessa, and analysis was performed using FlowJo (Treestar, Ashland, OR, USA). Antibodies used include anti-mouse CD11c (clone HL3; BD Biosciences; dilution: 1/200), anti-mouse MHC-II (clone M5/114.15.2; Tonbo Biosciences, San Diego, CA, USA; dilution: 1/150), anti-mouse CD3 (clone 500A2; BD Biosciences; dilution: 1/200), anti-mouse CD4 (clone RM4-5; BD Biosciences, dilution: 1/200), anti-mouse CD44 (clone IM7; eBioscience, dilution: 1/400), anti-mouse IL-17 (clone TC11-18H10; BD Biosciences, dilution: 1/100), anti-mouse IFN-γ (XMG1.2; BD Biosciences; dilution: 1/100), anti-mouse Thy1.1 (clone OX-7; BD Biosciences; dilution: 1/100) and anti-mouse CD103 (clone 2E7, eBioscience; dilution: 1/200).

### ELISpot

For analysis of production of antigen-specific cytokines by enzyme-linked immunospot (ELISpot) assay as before^4^, 96-well ELISpot plates were coated with monoclonal anti-mouse IL-17 antibody (clone 50101.111; R&D systems, Minneapolis, MN, USA) or anti-mouse IFN-γ antibody (clone R4-6A2; eBioscience) overnight, then blocked with media containing 10% fetal bovine serum. Total cells from lungs, or 5 × 10^5^ cells from LN were plated in the top well and subsequently diluted twofold in serial dilutions. Irradiated B6 splenocytes were used as APCs at a concentration of 1 × 10^6^ cells per well in the presence of 10 μg ml^−1^ peptide and 10 U ml^−1^ IL-2. After 24 h, plates were washed and probed with biotinylated anti-mouse IL-17 antibody (clone eBio17B7; eBioscience) or biotinylated anti-mouse IFN-γ antibody (clone XMG1.2; eBioscience), and binding detected using streptavidin–alkaline phosphatase (Vector Laboratories, Burlingame, CA, USA) with BCIP/NBT (Sigma Aldrich) as the substrate. Spots were visualized and enumerated using a CTL-Immunospot S5 MicroAnalyzer (C.T.L., Shaker Heights, OH, USA).

### Cell proliferation and mycobacterial killing assays

CD4^+^ T cells were isolated from single-cell suspensions generated from LNs and spleen from Ag85B TCR Tg mice on a Thy1.1 background using a CD4 L3T4 positive selection kit according to the manufacturer's instructions (Miltenyi Biotec Inc, San Diego, CA, USA). Naive CD4^+^ T cells (Th0) were intravenously transferred into B6 mice (on a Thy1.2 background) at 2 × 10^6^ cells per mouse. For assessment of proliferation *ex vivo*, whole lung cells isolated from mice receiving adoptively transferred T cells were labelled with 1 μM carboxyfluorescein succyinimidyl ester (CFSE, Invitrogen, Thermo Fisher) for 15 min at 37 °C at 1 × 10^7^ cells ml^−1^ in incomplete DMEM^3^. Cells were then cultured for 3 or 6 days with *Il10*^*−/−*^ BMDC stimulated overnight with zymosan alone (25 μg ml^−1^) or zymosan (25 μg ml^−1^)+Ag85B_240–254_ peptide (20 μg ml^−1^) in the presence or absence of *Mtb* HN878. Supernatants were collected and frozen at −80 °C. Cells were collected and T-cell proliferation was assessed by measuring carboxyfluorescein succyinimidyl ester dilution by flow cytometry. Transferred antigen-specific cells were tracked *in vivo* using Thy1.1 staining. Cell activation was measured by CD44 staining.

For the mycobacterial killing assay, BMDMs were infected overnight with *Mtb* HN878 at an multiplicity of infection of 1 in antibiotic-free cDMEM. The following day, infected BMDMs were co-cultured for 6 days at a 1:1 ratio with T cells previously activated with Ag85B-Z-DC as described above. Intracellular *Mtb* c.f.u. was assessed by lysing the BMDMs with 0.05% SDS before plating on 7H11 plates in serial dilutions.

### Cytokine and chemokine production

Cytokine and chemokine production in supernatants collected from stimulated BMDC or from T-cell-BMDC cultures was analysed using Milliplex Multiplex Assays (Millipore), using the protocol specified by the manufacturer. IL-10 (R&D; cat. no: DY417), IL-23 (R&D; cat. no: DY1887) and IL-6 (R&D; cat. no: DY406) were measured using a Duoset kit (R&D Systems), using the protocol specified by the manufacturer. IL-1β (BD; cat. no: 559603) was measured using an BD OptEIA IL-1β ELISA Set (BD Biosciences) using the protocol specified by the manufacturer.

### Immunofluorescence staining

Lung lobes were perfused with 10% formalin, fixed and paraffin embedded. For immunofluorescent staining, lung sections were cut, immersed in xylene to remove paraffin and then hydrated in 96% alcohol and phosphate-buffered saline. Antigens were unmasked with a DakoCytomation Target Retrieval Solution (Dako, Carpinteria, CA, USA), and non-specific binding was blocked with 5% (v/v) normal donkey serum and Fc block (BD Pharmingen). Endogenous biotin was neutralized by adding avidin, followed by incubation with biotin (Sigma Aldrich). Sections were probed with anti-B220 to detect B cells (clone RA3-6B2, BD Pharmingen; dilution: 1/100) and anti-CD3 to detect T cells (clone M-20, Santa Cruz Biotechnology, Santa Cruz, CA; dilution: 1/100). For analysis of B-cell follicles, follicles were outlined with the automated tool of the Zeiss Axioplan 2 microscope (Zeiss, Thornwood, NY, USA), and total area and average size was calculated in squared microns.

### Quantitative PCR with reverse transcription

Total RNA was isolated from lung tissue using an RNeasy RNA isolation kit (Qiagen, Valencia, CA, USA). Complementary DNA was generated using ABI reverse transcription reagents (ABI, Thermo Fisher) on a BioRad DNA Engine Thermal Cycler (BioRad, Hercules, CA, USA). Gene expression was assessed using primers from integrated DNA technologies (IDT) (iNOS (F-CAGCTGGGCTGTACAAACCTTC; R-CATTGGAAGTGAAGCGTTTCG;PROBE5′-/56-FAM/CGG GCA GCC TGT GAG ACC TTT GA/3BHQ_1/-3′);GAPDH (F-CTCGTCCCGTAGACAAAATGG; R-AATCTCCACTTTGCCACTGCA; PROBE5′-/56-FAM/CGG ATT TGG CCG TAT TGG GCG/3BHQ_1/-3′)) (Coralville, IA, USA) and ABI (CCL19: Mm00839967_g1; Saa3: Mm00441203-ml; Lcn2: Mm01324472_g1;Batf3: Mm01318274_m1; Itgae: Mm00434443_m1; Col1a1: Mm00801666_g1) and run on a Viia7 Real-Time PCR system (Life Technologies, Thermo Fisher). Expression of genes of interest was normalized to GAPDH expression, and log 10-fold induction over the control group was assessed using the ΔΔCT calculation.

### RNA-seq and gene set enrichment analysis

Total RNA was isolated from lung tissue using an RNeasy RNA isolation kit (Qiagen, Valencia, CA, USA). Each sample was assessed using Qubit 2.0 fluorometer (Invitrogen, Thermo Fisher) and Agilent Tapestation 2200 (Agilent Technologies, Santa Clara, CA, USA). Sequencing libraries were generated using Illumina TruSeq RNA Access library prep kit (Illumina, San Diego, CA, USA) following the manufacturer's protocol. Cluster generation and 75 bp single read single-indexed sequencing was performed on Illumina NextSeq 500 (Illumina). Sequencing analysis was done using mRNA-seq Analysis on Maverix Analytic Platform (Maverix Biomics, Inc, San Mateo, CA). Raw sequencing reads from Illumina sequencing platform that was converted into FASTQ file format were quality checked for potential sequencing issues and contaminants using FastQC. Adapter sequences, primers, Ns and reads with quality score <28 were trimmed using fastq-mcf of ea-utils and Trimmomatic. Reads with a remaining length of fewer than 20 bp after trimming were discarded. Single reads were mapped to the mouse genome (m10) using STAR in a strand specific manner. Cufflinks was used to determine fragments per kilobase of transcript per million mapped reads (FPKM) levels for each gene from the STAR alignment and was used as input for Cuffdiff. Pairwise differential expression was quantified using Cuffdiff. Read counts were then normalized across all samples and significant differentially expressed genes were determined by adjusted *P* value with a threshold of 0.05. For Gene Set Enrichment Analysis we have selected expressed genes from GSE11005 (top 10,000 based on average expression level). We then assembled ranked list using signed statistics values and performed pre-ranked GSEA using top 100 vaccine upregulated genes as a query gene set. The data shown is uncorrected for multiple comparisons. We also performed differential expression analysis using Limma between wild type and *Cd40l*^*−/−*^ groups at day 32 post-*P. murina* infection.

### Statistical analysis

Statistical analyses were performed using GraphPad Prism (La Jolla, CA, USA). For experiments with two groups, two-tailed Student's *t*-tests or Mann–Whitney test were performed. For two or more groups, a one-way analysis of variance or Kruskall–Wallis test was used. For multiple groups with multiple time points, a two-way analysis of variance was used. All plots show mean±s.d.

### Data availability

Sequence data that support the findings of this study have been deposited in BioProject with the primary accession code SRP091809. Other data that support the findings of this study are available from the corresponding author upon request.

## Additional information

**How to cite this article:** Griffiths, K. L. *et al*. Targeting dendritic cells to accelerate T-cell activation overcomes a bottleneck in tuberculosis vaccine efficacy. *Nat. Commun.* **7,** 13894 doi: 10.1038/ncomms13894 (2016).

**Publisher's note:** Springer Nature remains neutral with regard to jurisdictional claims in published maps and institutional affiliations.

## Supplementary Material

Supplementary Information Supplementary Figures and Supplementary Tables
