# Supplementary material for: Targeting dendritic cells to accelerate T-cell activation overcomes a bottleneck in tuberculosis vaccine efficacy
Source: Nat Commun. 2016 Dec 22;7:13894. doi: 10.1038/ncomms13894 (PMC5192216; doi:10.1038/ncomms13894)
Supplement: Supplementary Information — Supplementary Figures and Supplementary Tables [file ncomms13894-s1.pdf]

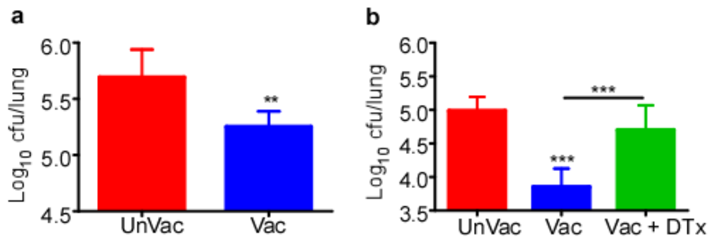

Supplementary Figure 1. Antigen availability or Tregs are not a TB vaccine bottleneck. **(a)** Mice were vaccinated with BCG s.c. followed by mucosal boost with Ag85B$_{240-254}$ peptide in mucosal adjuvant, rested for 4 weeks and then infected with 1000 cfu *Mtb* HN878. Bacterial burden in the lungs was determined at 30 *dpi*. **(b)** Foxp3.DTR mice were vaccinated parentally with BCG followed by mucosal boost with Ag85B$_{240-254}$ peptide and rested for 4 weeks, following which they were infected with *Mtb* HN878 (~100cfu). Foxp3^{+} cells were depleted using i.p. administration of diphtheria toxin (DTx). Bacterial burden in the lungs was assessed at 30 *dpi*. n = 5 biological replicates, **p ≤ 0.01, ***p ≤ 0.001 using student's *t-test* (a) one way ANOVA (b).

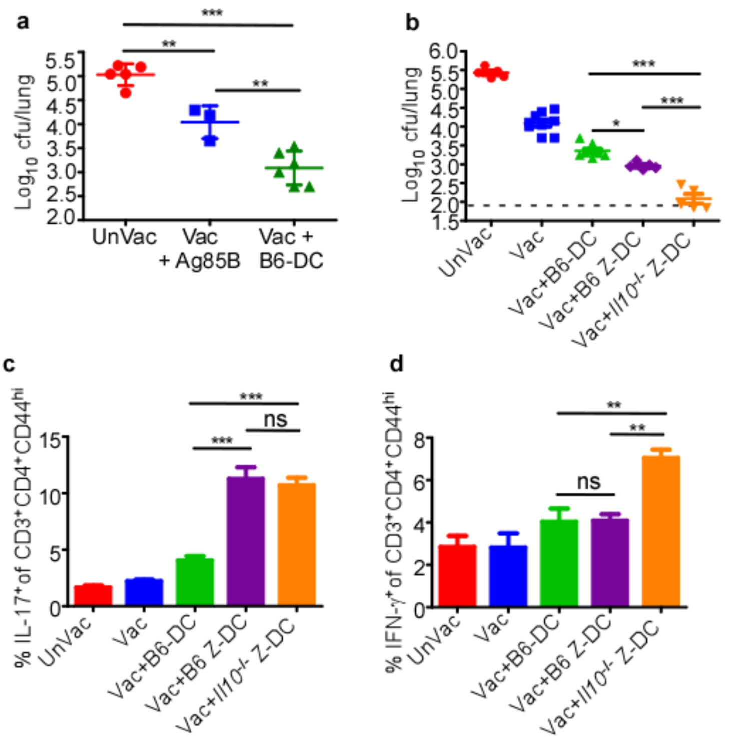

Supplementary Figure 2. Il10-/- Z-DC transfer results in superior vaccine-mediated *Mtb* control. B6 mice were vaccinated with BCG s.c. followed by $Ag85B_{240\text{-}254}$ boost in mucosal adjuvant. Mice were rested for a further 4 weeks before being infected with ~100 cfu *Mtb* HN878 via aerosol. Mice received i.t. transfer of either $Ag85B_{240\text{-}254}$ antigen alone (20µg/mL), B6 DCs pulsed with Ag85B (20µg/mL), or B6 or *Il10*$^{-/-}$ DCs activated with Zymosan (25µg/mL) as well as pulsed with Ag85B (20µg/mL). Lungs were harvested at 20 *dpi*. **(a-b)** Lung bacterial burden was determined by plating. Flow cytometry was used to assess the frequency of **(c)** IL-17 and **(d)** IFN-γ production by Ag85B-specific $CD4^+CD44^{hi}$ T cells. n = 3-8 biological replicates ± SD. $^*p \le 0.05$, $^{**}p \le 0.01$, $^{***}p \le 0.001$ by one way ANOVA. Dotted lines represent limit of detection by plating. ns-not significant.

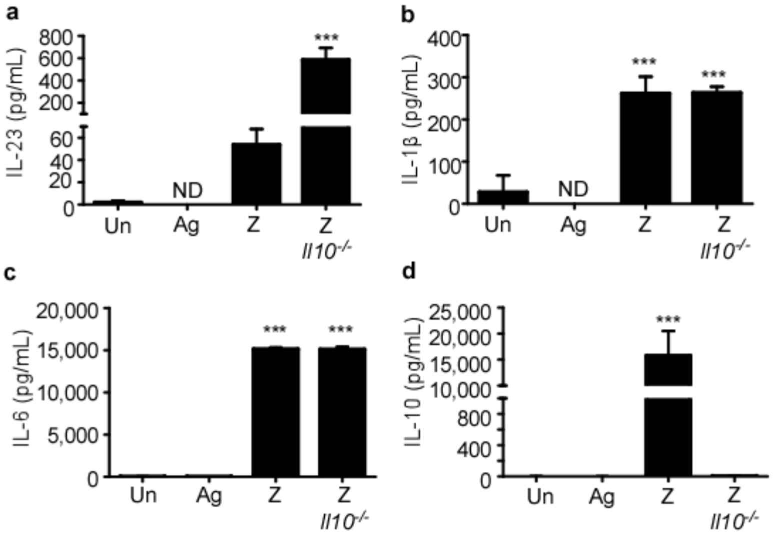

Supplementary Figure 3. Zymosan induces cytokines in DCs. BMDC generated from B6 or *Il10*$^{-/-}$ mice were stimulated overnight with either Ag85B antigen alone (Ag), or Zymosan (Z) along with Ag85B antigen. Supernatants were collected and cytokine concentrations were assessed by Milliplex assay (IL-1β and IL-6) or ELISA (IL-23, IL-10). n = 3 technical replicates *p ≤ 0.05, **p ≤ 0.01, ***p ≤ 0.001 by one way ANOVA. ND = not detected. Where ND is not annotated, analyte was detected, but is not visible clearly on the scale used.

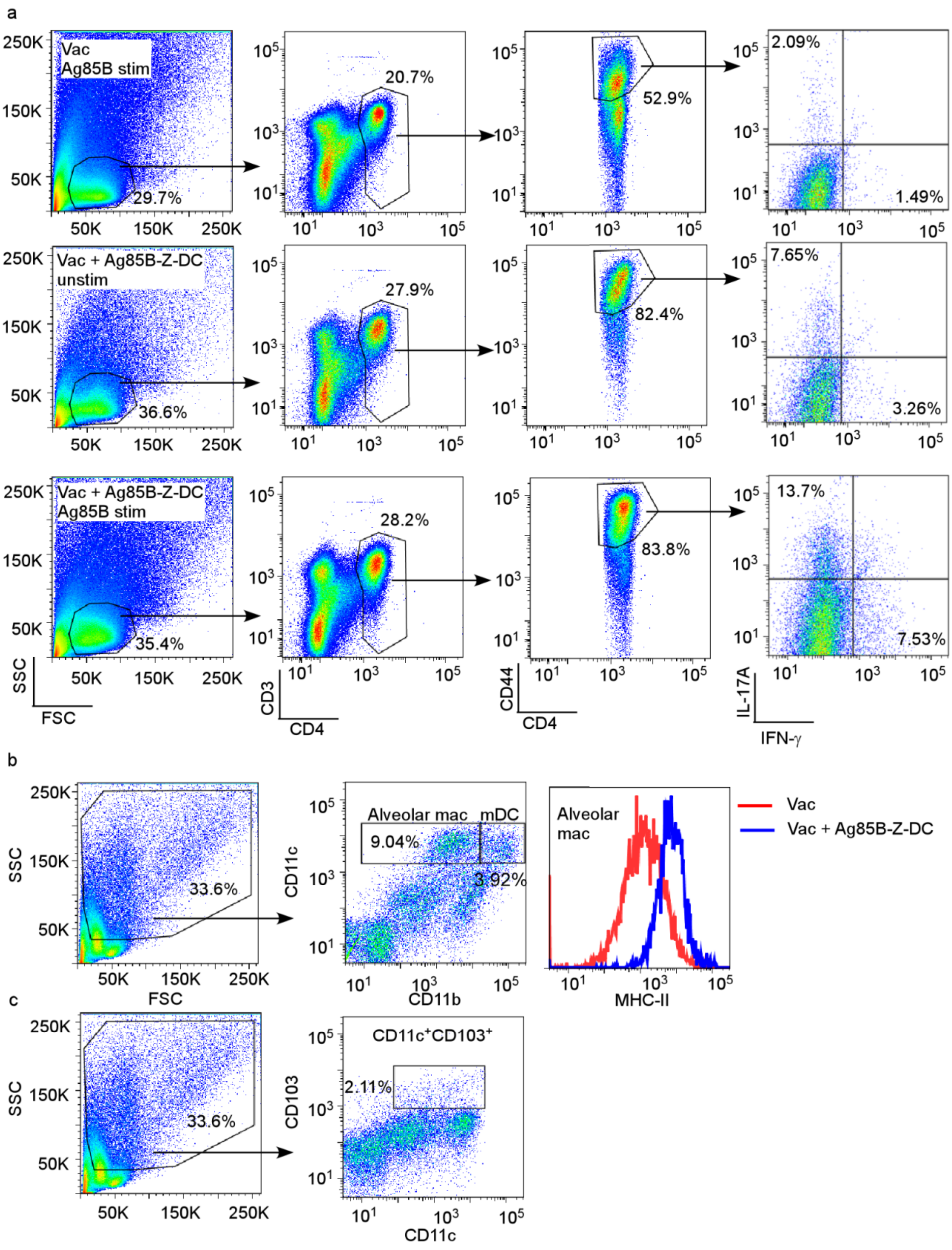

Supplementary Figure 4. Gating strategy for T cells and myeloid cells. Lungs were harvested from vaccinated *Mtb*-infected mice, with or without Ag85B-Z-DC transfer and single cell suspensions were prepared and flow cytometry was carried out**. (a)** Lymphocytes were gated based on forward and side scatter, followed by gating on $CD3^{+}CD4^{+}$ cells, then $CD44^{hi}$ cells, and finally on either IL-17 or IFNγ- cytokine-producing cells. Top panel shows representative plots from a vaccinated *Mtb*-infected mouse following restimulation with Ag85B peptide. Middle panel shows representative plots from a vaccinated Z-DC-recipient

Mtb-infected mouse cultured without restimulation with antigen. Bottom panel shows representative plots from a vaccinated Z-DC-recipient *Mtb*-infected mouse following restimulation overnight with Ag85B peptide as described under methods. **(b)** Large lung cells were gated for, followed by gating on $CD11c^{+}CD11b^{-}$ alveolar macrophages or myeloid DCs (mDCs). MHC-II MFI was calculated for alveolar macrophage subset. **(c)** $CD11c^{+}CD103^{+}$ cells were gated from large lung cells.

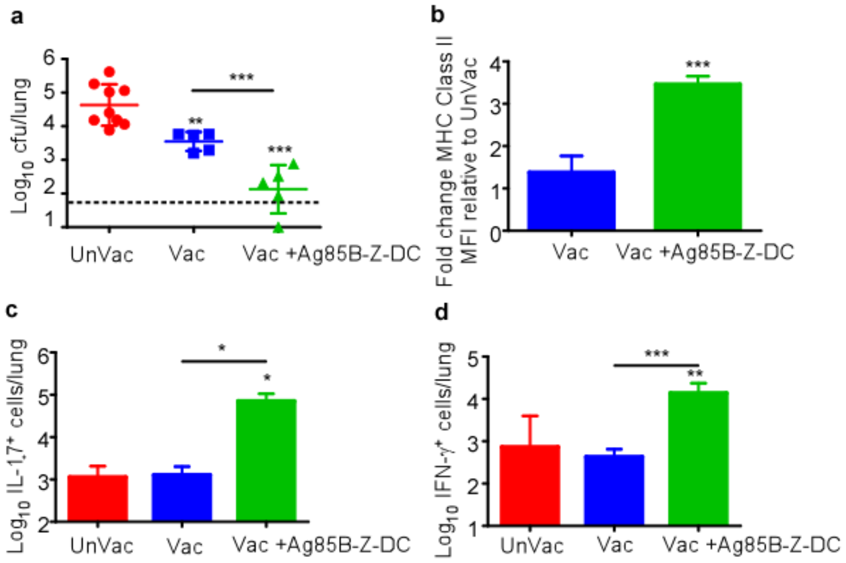

Supplementary Figure 5. Z-DC transfer confers superior vaccine protection in *Mtb* H37Rv infection. B6 mice were vaccinated with BCG s.c. followed by $Ag85B_{240\text{-}254}$ boost in mucosal adjuvant and rested further for 4 weeks. Mice received Z-DC transfer i.t. on -1 and 4 *dpi*, following infection with *Mtb* H37Rv (~100cfu). Lungs were harvested on 20 *dpi*. **(a)** Lung bacterial burden was determined by plating. Flow cytometry was used to assess **(b)** MFI of MHC-II expression on alveolar macrophages, and fold change MFI relative to UnVac was calculated. **(c)** IL-17 and **(d)** IFN-γ production by Ag85B-specific $CD4^+CD44^{hi}$ lung T cells was assessed by flow cytometry. n = 5-9 biological replicates ± SD. *$p \leq 0.05$, **$p \leq 0.01$, ***$p \leq 0.001$ by one way ANOVA. Dotted line represents limit of detection by plating.

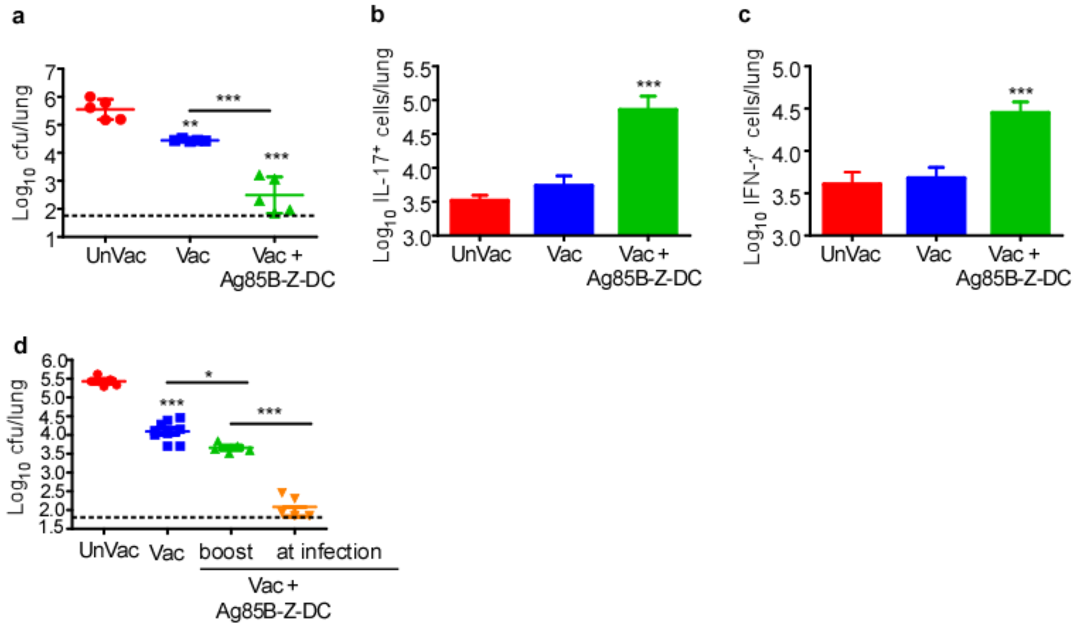

Supplementary Figure 6. Z-DC transfer induces durable and superior vaccine Mtb control. B6 mice were vaccinated with BCG s.c. followed by Ag85B$_{240-254}$ boost in mucosal adjuvant and rested for 10 weeks following the final vaccination before challenge with *Mtb* HN878 (~100cfu) via aerosol. Vaccinated mice received Ag85B-Z-DC on -1 and 4 *dpi*. Lungs were harvested at 20 *dpi*. **(a)** Bacterial burden was determined by plating. Flow cytometry on lung single cell suspensions was used to determine Ag85B-specific **(b)** IL-17 and **(c)** IFN-γ production by CD4^{+}CD44^{hi} T cells. **(d)** B6 mice were vaccinated with BCG s.c. and rested for 4 weeks before receiving Ag85B$_{240-254}$ mucosal boost as a means of vaccination. Mice were rested for a further 4 weeks before infection with *Mtb* HN878 (~100cfu). A control vaccinated group received Z-DC transfer at -1 and 4 *dpi.* Lungs were harvested at 20 *dpi*. Control groups for this experiment are the same as the groups shown in Supplementary Fig. 2b (i.e. UnVac, Vac and Vac+Z-DC at infection). **(d)** Bacterial burden was determined by plating. n = 5-8 biological replicates ± SD. *$p \leq 0.05$, **$p \leq 0.01$, ***$p \leq 0.001$ by one way ANOVA (a,d) or Kruskall-Wallis test (b,c). Dotted lines on bacterial burden plots represent the limit of detection by plating.

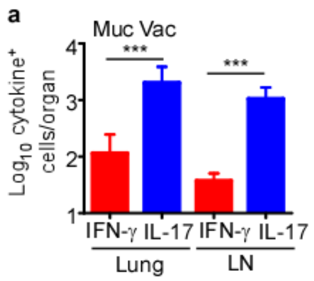

Supplementary Figure 7. Mucosal vaccination induces IL-17-producing mucosal T cells. B6 mice were vaccinated with either **(a)** three doses of Ag85B in mucosal adjuvant, LT-IIb i.n., or **(b)** BCG s.c. Two weeks post-vaccination, lungs and draining LN were harvested and assessed for Ag85B-specific IL-17 and IFN-γ responses by antigen-driven ELISpot. n = 5 biological replicates ± SD, *p ≤ 0.05, **p ≤ 0.01, ***p ≤ 0.001 by student's *t-test*

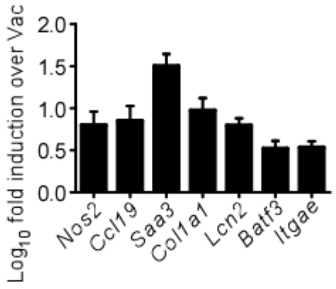

Supplementary Figure 8. Gene upregulation in vaccinated mice receiving Z-DC transfer. B6 mice were vaccinated with BCG s.c. followed by the final vaccination before challenge with *Mtb* HN878 (~100cfu) via aerosol. Vaccinated mice received Ag85B-Z-DC on -1 and 4 *dpi*. Lungs were harvested and RNA was isolated at 8 *dpi*, and mRNA expression of specific genes upregulated in Supplementary Table 1 were assessed using RT-PCR. n=5 biological replicates ± SD.

Supplementary Table 1. Z-DC transfer in vaccinated mice induces early differential gene expression in the lung. B6 mice vaccinated parenterally with BCG followed by mucosal Ag85B were rested for 4 weeks, and infected with *Mtb* HN878 (100cfu) with or without Ag85B-Z-DC transfer. RNA-Seq was performed on RNA isolated at 8 *dpi* from lungs of vaccinated mice and vaccinated mice receiving Z-DC transfer. The top 100 genes upregulated in mice receiving Z-DC transfer compared to vaccinated mice are shown. Log fold change was calculated by running CuffQuant and CuffDiff with default parameters. n = 4-5, q value is comparing Vac + Ag85B-Z-DC to Vac and was calculated using the Benjamini Hochberg procedure.

Gene	Vac	Vac + Ag85B-Z-DC	Log_2 Fold change (Vac + Z-DC over Vac)	q value
**Top 100 upregulated**				
Itln1	0	10.7163	NA	0.000178
Capn9	0	0.204714	NA	0.000178
Krt16	0	1.19234	NA	0.000178
Npy	0	5.9684	NA	0.000178
Gif	0	0.207858	NA	0.000178
Krt6a	0	0.2442	NA	0.000178
Saa2	0	0.356234	NA	0.000178
Krtap10-4	0	1.37429	NA	0.000178
Lce1a1	0	0.738693	NA	0.000178
B4galnt4	0.0190237	1.6197	6.411784943	0.003029
H2-M2	2.68413	213.949	6.316668579	0.000178
Clca3	0.504213	35.4305	6.1348147	0.000178
AA467197	0.244276	13.5729	5.796073065	0.00149
Crmp1	0.249939	11.7662	5.55692862	0.000178
5430421N21Rik	0.760478	34.3881	5.498859084	0.000178
Chl1	0.0224995	0.822516	5.192078897	0.000178
Pdcd1lg2	0.0974999	3.54616	5.184713082	0.000178
Htr7	0.0281838	1.0007	5.149999581	0.000339
Mmp13	0.0881548	2.56757	4.864220673	0.000178
Col24a1	0.044972	1.19391	4.730523231	0.000178
Slc6a12	0.104625	2.59068	4.630031297	0.000178
Saa3	24.039	587.175	4.610341757	0.000178
Fcrlb	0.13447	3.08917	4.521863015	0.001076
Ctla4	0.441005	9.63227	4.449008915	0.000178
Nos2	0.0658889	1.43302	4.442879493	0.000178
Trat1	0.146703	3.16573	4.431567937	0.001623
Fcgbp	0.573255	12.287	4.421811866	0.000178
Irg1	0.799041	16.3546	4.355283132	0.000178
Tnfrsf25	0.0539148	1.06124	4.298925792	0.00149
D830030K20Rik	0.0189993	0.372175	4.291962978	0.016624
Slc26a4	1.21492	23.5389	4.27611368	0.000178
Slc7a11	0.828555	15.5216	4.227536003	0.000178
Duox1	0.01998	0.355212	4.152051832	0.001888
St8sia1	0.10231	1.75855	4.103367286	0.000178
Acsbg1	0.076619	1.28121	4.063660957	0.000178
Cebpe	0.290194	4.83823	4.059389758	0.000178

Inhba	2.83292	46.0799	4.023775819	0.000178
Matk	0.351055	5.66538	4.012403745	0.000178
Cyp21a1	0.251479	3.94212	3.970461857	0.000178
Aqp3	0.289661	4.5221	3.964555538	0.000178
Neil3	0.174708	2.66609	3.931707906	0.000178
Gfi1	0.0641291	0.922536	3.846554145	0.007762
Dcstamp	0.0246029	0.344911	3.809323856	0.006746
Thbs4	0.0570812	0.797282	3.804002529	0.000178
Col6a5	0.0297324	0.408223	3.779249641	0.000178
Bhlhe22	0.0172607	0.225313	3.706367677	0.006746
Cd3g	0.101908	1.30656	3.680434162	0.006075
Mettl21e	0.0227752	0.287978	3.660422973	0.006185
Cd6	0.740617	9.07419	3.614969298	0.000178
Sit1	0.0734771	0.89003	3.598487373	0.005059
Ksr2	0.0939316	1.13507	3.595026878	0.000178
Gpnmb	0.0577296	0.687538	3.574056319	0.000178
Gpr176	0.0470191	0.559073	3.571717843	0.000178
Stag3	0.0180327	0.211966	3.555145541	0.005059
Adh6-ps1	0.101677	1.17639	3.532301151	0.000178
Camk1g	0.0309649	0.358071	3.531539988	0.008196
G630071F17Rik	0.231009	2.62728	3.507548998	0.000178
Cdkn2a	0.24118	2.70985	3.490030814	0.013485
Chrna2	0.495547	5.49907	3.47209385	0.000178
Shcbp1	0.128231	1.41412	3.463087568	0.000178
Ttbk1	0.167005	1.80966	3.437755467	0.000178
Pif1	0.0494123	0.530392	3.424116899	0.000178
Slamf8	0.0484941	0.519574	3.4214481	0.002902
Ern2	0.0641069	0.679673	3.406289261	0.000178
Ngfr	0.0447086	0.467413	3.386073585	0.007086
Glrp1	0.251305	2.58243	3.361217963	0.000178
Psd2	0.050369	0.511807	3.344991885	0.000178
Cd4	0.422189	4.26867	3.33782574	0.000178
BC021785	0.168975	1.6675	3.302805042	0.000178
Ctss	0.95682	9.34217	3.287438247	0.000178
Cenpe	0.0946403	0.919392	3.280153561	0.000178
Cenpf	0.0771171	0.737101	3.256739611	0.000178
Rhox8	3.36763	32.0348	3.24983444	0.000178
Ccnb1	0.103746	0.986362	3.249061507	0.000178
Nlrc3	0.166393	1.58175	3.248854949	0.000178
Spag5	0.117542	1.10838	3.237204327	0.000178
Mx1	0.434306	4.03685	3.216446192	0.000178
Gcm1	0.255357	2.36372	3.210471635	0.000178
Mt3	1.16952	10.8238	3.210218648	0.000178
Trpm2	0.421391	3.87631	3.201452545	0.000178
Cd3e	31.6042	290.1	3.198362094	0.000178
Stac2	1.25	11.4355	3.193519447	0.000178
Iqgap3	0.362616	3.2791	3.17678541	0.000178
P4ha3	0.108706	0.981932	3.175191548	0.000178
Ltb4r1	0.301006	2.70622	3.168414977	0.000178
Aif1	1.20686	10.8119	3.16328984	0.000178
Egln3	0.873409	7.765	3.152256622	0.000178
Agr2	0.878205	7.77502	3.146216733	0.000178
Gm20753	0.540132	4.75825	3.139047145	0.000178
Piwil2	0.121858	1.07235	3.137502986	0.000178

Lcn2	0.605478	5.31364	3.133554043	0.000178
Ccnf	0.484787	4.17898	3.107727937	0.000178
Tnf	0.0475171	0.409095	3.105917209	0.001076
Pigr	2.19756	18.6484	3.085077394	0.000178
Batf3	0.661354	5.55094	3.069237491	0.000178
Fam25c	1.31578	10.8761	3.047171127	0.010602
Oit3	0.0290683	0.239953	3.045233244	0.007762
Adam8	0.0718939	0.590792	3.038709018	0.000178
Mex3a	0.132881	1.08242	3.026053661	0.000178
Scin	0.270218	2.19299	3.020703548	0.000178

Supplementary Table 2. DC transfer in vaccinated mice upregulates genes in the T cell signalling and APC activation pathways. RNA-Seq was performed on RNA isolated at 8 *dpi* from lungs of B6 mice vaccinated parenterally with BCG followed by mucosal Ag85B with or without Z-DC transfer as in Supplementary Table 1. Ingenuity Pathway Analysis was performed to identify top pathways upregulated in mice receiving Z-DC transfer compared to vaccinated mice. For each network, a Consistency Score was calculated that rewards for paths from regulator->target->disease or function that are consistent. n = 4-5, p values were calculated using Fisher's Exact Test.

**Top canonical pathway**		
Name	*p-value*	*Overlap*
T cell receptor signaling	5.51E-09	13.40%
CD28 signaling in T helper cells	7.14E-09	11.90%
iCOS-iCOSL signaling in T helper cells	2.08E-08	12.00%
Role of NFAT in regulation of the immune response	1.26E-07	8.80%
OX40 signaling pathway	1.96E-07	12.40%
**Upstream regulators**		
Upstream regulator	*p-value of overlap*	*Predicted activation*
Lipopolysaccharide	8.65E-22	Activated
CSF2	3.54E-15	Activated
IL6	2.51E-14	Activated
PTGER2	1.47E-13	Activated
**Top diseases and bio functions**		
Name	*p-value*	*#Molecules*
Inflammatory response	5.10e-04 - 2.36e-17	123
Immunological disease	4.80e-04 - 7.67e-10	100
Respiratory disease	5.49e-04 - 8.88e-10	73
Connective tissue disorders	4.80e-04 - 1.27e-09	71
Inflammatory disease	4.80e-04 - 1.27e-09	87
Hematological system development and function	5.49e-04 - 1.05e-22	129
Tissue morphology	4.45e-04 - 1.05e-22	120
Immune cell trafficking	4.91e-04 - 2.64e-15	74
Lymphoid tissue structure and development	4.40e-04 - 8.05e-15	65
Cell morphology	4.45e-04 - 9.48e-18	104
Cellular function and maintenance	5.10e-04 - 1.47e-15	128
Cell-to-cell signaling and interaction	5.10e-04 - 2.64e-15	87
Cellular development	5.49e-04 - 5.08e-15	144
Cellular growth and proliferation	3.96e-04 - 6.24e-13	144
**Top regulator effect networks**		
ID regulators	*Diseases & functions*	*Consistency score*
Alpha catenin,CD44,CXCL12,ERK,F2,IL12 (complex)	Activation of myeloid cells	23.597

CBX5,ERK,FOXA2,Ifn,IL5,Interferon alpha,Jnk	Activation of myeloid cells	23.094
CBX5,CEBPB,CHUK,ERK,Ifn,IL10RA,IL12 (complex)	Activation of macrophages	22.738
Akt,CXCL12,Ifn,IL10RA,IL12 (complex),IL17A,IL18	Activation of myeloid cells	21.442
CXCL12,ERK,F2,HMGA1,IKBKB,IKBKG,IL10RA	Arteriosclerosis, binding of leukocytes	18.276

Top networks

ID associated network functions	*Score*
Inflammatory response, cell-to-cell signaling and interaction, hematological system development and function	39
Hematological system development and function, cellular assembly and organization, DNA replication, recombination and repair	37
Immunological disease, inflammatory disease, inflammatory response	34
Cell-mediated immune response, cellular development, cellular function and maintenance	33
Embryonic development, organismal development, organ morphology	33
